# Quantitative functional profiling of *ERCC2* mutations deciphers cisplatin sensitivity in bladder cancer

**DOI:** 10.1172/JCI186688

**Published:** 2025-08-15

**Authors:** Judit Börcsök, Diyavarshini Gopaul, Daphne Devesa-Serrano, Clémence Mooser, Nicolas Jonsson, Matteo Cagiada, Dag R. Stormoen, Maya N. Ataya, Brendan J. Guercio, Hristos Z. Kaimakliotis, Gopa Iyer, Kresten Lindorff-Larsen, Lars Dyrskjøt, Kent W. Mouw, Zoltan Szallasi, Claus S. Sørensen

**Affiliations:** 1 Biotech Research & Innovation Centre, University of Copenhagen, Copenhagen, Denmark.; 2 Translational Cancer Genomics Group, Danish Cancer Institute, Copenhagen, Denmark.; 3 Linderstrøm-Lang Centre for Protein Science, Department of Biology, University of Copenhagen, Copenhagen, Denmark.; 4 Department of Oncology, Copenhagen University Hospital, Rigshospitalet, Copenhagen, Denmark.; 5 Department of Urology, Indiana University School of Medicine, Indianapolis, Indiana, USA.; 6 Genitourinary Oncology Service, Department of Medicine, Memorial Sloan Kettering Cancer Center, New York, New York, USA.; 7 James P. Wilmot Cancer Institute, University of Rochester, Rochester, New York, USA.; 8 Department of Clinical Medicine, Aarhus University, Aarhus, Denmark.; 9 Department of Molecular Medicine, Aarhus University Hospital, Aarhus, Denmark.; 10 Department of Radiation Oncology, Dana-Farber Cancer Institute, Boston, Massachusetts, USA.; 11 Department of Radiation Oncology, Brigham & Women’s Hospital, Boston, Massachusetts, USA.; 12 Harvard Medical School, Boston, Massachusetts, USA.; 13 Computational Health Informatics Program, Boston Children’s Hospital, Boston, Massachusetts, USA.; 14 Department of Bioinformatics, Semmelweis University, Budapest, Hungary.

**Keywords:** Cell biology, Genetics, Oncology, Cancer, DNA repair, Urology

## Abstract

Tumor gene alterations can serve as predictive biomarkers for therapy response. The nucleotide excision repair (NER) helicase ERCC2 carries heterozygous missense mutations in approximately 10% of bladder tumors, and these may predict sensitivity to cisplatin treatment. To explore the clinical actionability of ERCC2 mutations, we assembled a multinational cohort of 2,012 individuals with bladder cancer and applied the highly quantitative CRISPR-Select assay to functionally profile recurrent ERCC2 mutations. We also developed a single-allele editing version of CRISPR-Select to assess heterozygous missense variants in their native context. From the cohort, 506 ERCC2 mutations were identified, with 93% being heterozygous missense variants. CRISPR-Select pinpointed deleterious, cisplatin-sensitizing mutations, particularly within the conserved helicase domains. Importantly, single-allele editing revealed that heterozygous helicase-domain mutations markedly increased cisplatin sensitivity. Integration with clinical data confirmed that these mutations were associated with improved response to platinum-based neoadjuvant chemotherapy. Comparison with computational algorithms showed substantial discrepancies, highlighting the importance of precision functional assays for interpreting mutation effects in clinically relevant contexts. Our results demonstrate that CRISPR-Select provides a robust platform to advance biomarker-driven therapy in bladder cancer and supports its potential integration into precision oncology workflows.

## Abstract 2

<div> <div> <div> We used CRISPR-Select, a highly quantitative functional assay, to map the landscape of cisplatin-sensitizing ERCC2 mutations in bladder cancer. </div> </div> </div>

## Introduction

Platinum-based chemotherapy is approved for use in muscle-invasive and metastatic bladder cancer. For muscle-invasive bladder cancer (MIBC), neoadjuvant cisplatin-based chemotherapy prior to radical cystectomy improves overall survival (OS) compared with radical cystectomy alone (1, 2). In the metastatic setting, cisplatin or carboplatin combinations have been a cornerstone of therapy for decades. However, tumor response to platinum-based chemotherapy varies markedly across patients, and reliable predictive biomarkers are needed to improve patient stratification and inform therapy selection.

Loss of DNA repair pathway function occurs in a subset of tumors and has the potential to be exploited therapeutically. Approximately 10% of bladder tumors harbor somatic missense mutations in the nucleotide excision repair (NER) gene *ERCC2* (3–5). The NER pathway primarily repairs bulky intrastrand adducts including UV- and platinum-induced lesions (6, 7). The *ERCC2* gene encodes a DNA helicase (also known as XPD) that unwinds the DNA duplex near the damage site and verifies the lesion. In a subset of retrospective clinical-genomic studies of MIBC, patients with somatic *ERCC2* mutations were more likely to experience a pathologic complete response (pCR) following cisplatin-based neoadjuvant chemotherapy than patients lacking a tumor *ERCC2* mutation (8, 9). Importantly, this improvement in pCR rate translated to an OS benefit for patients with an *ERCC2* mutation (9). However, *ERCC2* missense mutations have not been found to be significantly associated with response to platinum-based chemotherapy in all studies (10–12), highlighting the complex nature of biological mechanisms underlying treatment response.

Functional analysis of a subset of clinically observed *ERCC2* mutations suggests that the majority are sufficient to confer NER deficiency and increased cisplatin sensitivity (13). However, the complementation-based approaches used to profile *ERCC2* mutant alleles to date have several limitations, including the inability to express mutant proteins at physiologically relevant levels, as well as limitations of scalability and quantitative sensitivity. CRISPR-Select (14, 15) is a recently developed CRISPR-based editing approach with inbuilt controls generated in the same culture system as mutations of interest. These editing controls ensure high accuracy and precision as they eliminate common concerns with functional assays, such as overexpression or subcloning artifacts.

Here, we assembled clinical and genomic data from patients with bladder cancer, which is, to our knowledge, the largest cohort of *ERCC2*-mutant bladder tumors characterized to date. We comprehensively mapped the mutational landscape of *ERCC2* and leveraged CRISPR-Select to decode the functional impact of prevalent variants revealing a marked cisplatin sensitivity of the most common helicase domain variants. Moreover, we developed single allele CRISPR-Select to allow functional assessment of *ERCC2* mutations in a heterozygous state. Finally, we compared *ERCC2* mutation impact scores between experimental CRISPR-Select determination and computational methods. In conclusion, our findings set the stage for integrated clinical interpretation of *ERCC2* mutation status for optimized bladder cancer treatment.

## Results

### Assembly and characterization of a large multiinstitutional ERCC2-mutant bladder cancer cohort.

The *ERCC2* mutation frequency in several reported bladder cancer cohorts ranges between 8% to 20% (3–5). To interrogate the nature of *ERCC2* mutations more deeply in bladder cancer, we assembled a multiinstitutional cohort of bladder cancer cases (*n* = 2,012; Supplemental Figure 1A; supplemental material available online with this article; https://doi.org/10.1172/JCI186688DS1) that represents the largest clinically and/or genomically annotated database of *ERCC2*-mutant cases analyzed to date. The complete cohort consists of 675 patient-derived tumor samples analyzed by whole-exome sequencing (WES) and 1,337 samples analyzed by targeted panel sequencing. Cases with available sequencing data and clinical information were divided into 2 clinically distinct groups: a neoadjuvant cohort and a metastatic cohort. The neoadjuvant cohort consisted of 284 tumors collected from 5 cohorts of nonmetastatic MIBC patients who received cisplatin-based neoadjuvant chemotherapy (NAC): Dana-Farber Cancer Institute and Memorial Sloan Kettering Cancer Center (DFCI-MSKCC, *n* = 50) (5), Philadelphia (*n* = 48) (9, 16), Aarhus (*n* = 60) (12), MSK IMPACT (*n* = 38), and Indiana (*n* = 88) (Supplemental Figure 1A). The metastatic cohort was comprised of 429 tumors collected from 3 cohorts of patients: Aarhus (*n* = 105) (12), DFCI Oncopanel (*n* = 132) (17), and Urothelial Cancer – Genomics Analysis to Improve Patient Outcomes and Research (UC-GENOME, *n* = 192) (18) (Supplemental Figure 1A). Of the 429 patients in the metastatic cohort, 322 patients received platinum-based chemotherapy. In the DFCI Oncopanel and UC-GENOME cohorts, the primary tumor was sequenced in 77% and 87% of the patients, respectively, whereas tumor from a metastatic site was sequenced in 19% and 13%, respectively (the remaining 4% of samples in the DFCI Oncopanel cohort were derived from locally recurrent sites or the information was not available). In the Aarhus cohort, primary tumor specimens were sequenced in all 165 cases. In addition to the assembled neoadjuvant and metastatic cohorts, bladder cancer cases from The Cancer Genome Atlas (TCGA) cohort (4) were analyzed separately and consisted of 412 muscle-invasive, high-grade urothelial tumors analyzed by WES.

**Figure 1:**
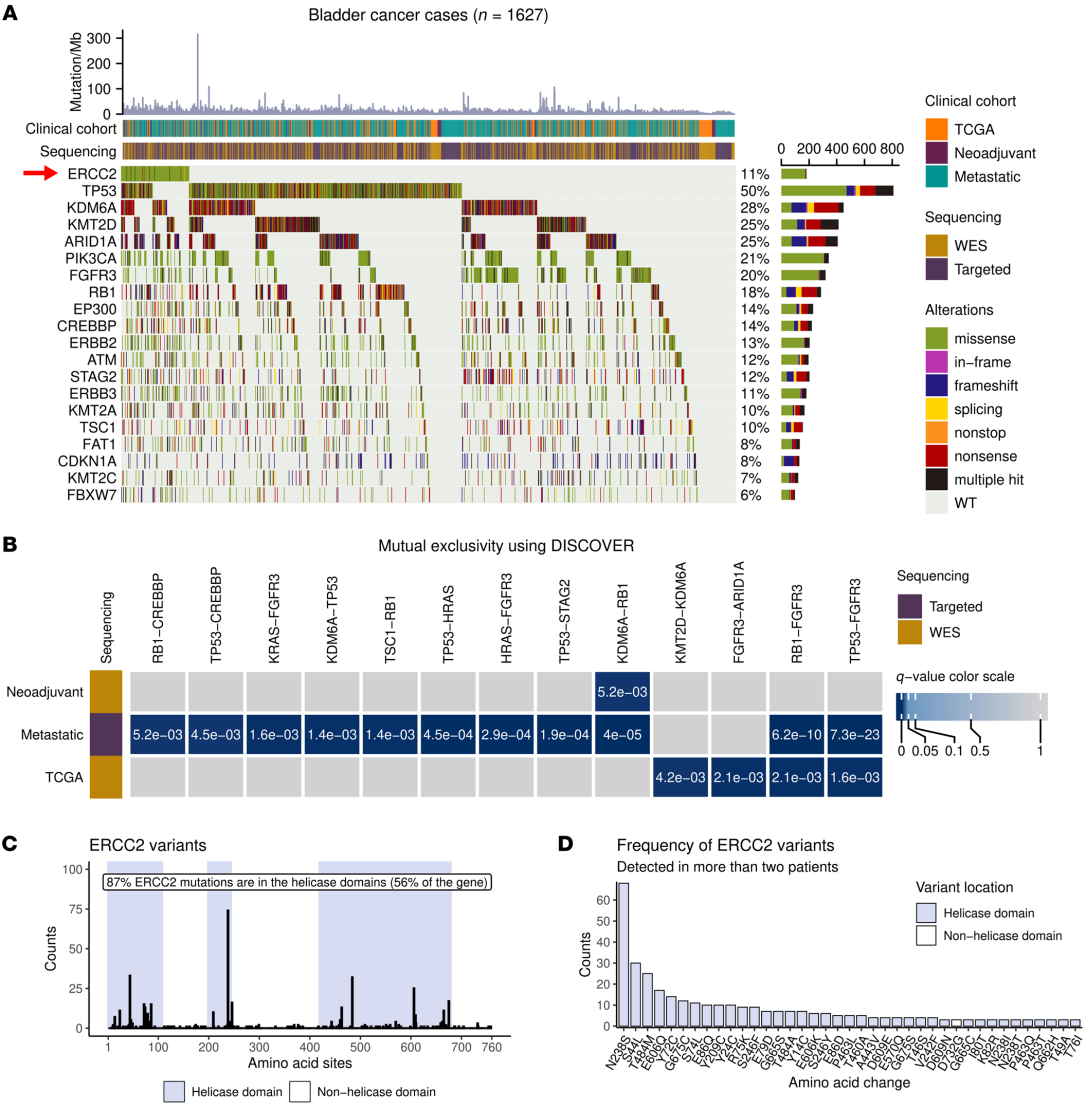
Extensive analysis of MIBC cohorts. (**A**) Mutation landscape of the bladder cancer cases analyzed in the neoadjuvant, metastatic, and TCGA patient cohorts. (**B**) Mutually exclusive gene pairs identified in the neoadjuvant, metastatic, and TCGA cohorts using the DISCOVER test. No cooccurring gene pairs were detected using the DISCOVER test. Targeted and whole-exome sequencing (WES) cohorts were analyzed separately. (**C**) 87% of somatic small-scale mutations in *ERCC2* occur in the helicase domains of the gene, although the helicase domains only constitute 56% of the gene. The observed ratio of helicase-domain variants was compared with an expected ratio of variants occurring randomly along the gene (χ^2^ test: *P* = 6.12 × 10^–30^). (**D**) The most frequent *ERCC2* variants that were detected in the collected cohorts.

We performed comprehensive mutational analyses for all tumors across the 3 cohorts (neoadjuvant, metastatic, and TCGA). Somatic mutations, including single-nucleotide variants (SNVs) as well as short insertions and deletions (indels) identified by WES or targeted panel sequencing, were annotated. We focused our analyses on nonbenign exonic and splice site mutations affecting a gene identified to be significantly mutated in MIBC (4). The 20 most frequently mutated genes are shown in Figure 1A and Supplemental Figure 1, B and C.

In the neoadjuvant cohort, the median nonsynonymous mutation rate was 5 mutations per megabase (Mb) for WES cases, *TP53* was the most frequently mutated gene (57%), and *ERCC2* was mutated in 19% of the cases (Supplemental Figure 1B). The Indiana and MSK-IMPACT targeted sequencing cohorts were excluded from Figure 1A and Supplemental Figure 1B because the Indiana cohort only had mutational data for *ERCC2* and *TP53*, and the MSK-IMPACT cohort consisted exclusively of *ERCC2-*mutant cases (Supplemental Figure 1C). However, even with these 2 cohorts excluded, the frequency of *ERCC2* mutations in the neoadjuvant cases summarized in Supplemental Figure 1B may still be higher than in a nonselected MIBC population because patients in the DFCI-MSKCC and Philadelphia cohorts were specifically included in the cohorts based on tumor response to cisplatin-based therapy. We performed mutual exclusivity and cooccurrence analyses (Methods) for mutations in genes significantly mutated in BLCA using Discrete Independence Statistic Controlling for Observations with Varying Event Rates (DISCOVER) (19). There were no genes with mutations that significantly cooccurred or were mutually exclusive with *ERCC2* mutations; however, we did identify a mutually exclusive relationship between *RB1* and *KDM6A* in the subset of the neoadjuvant cohort with available WES data (Figure 1B) in agreement with previous reports (20).

In the metastatic cohort, the median nonsynonymous mutation rate was 4 and 11 mutations per Mb for WES and panel sequencing samples, respectively. *TP53* was mutated in 50% of cases, and *ERCC2* was mutated in 11% of cases (Supplemental Figure 1B). We identified several mutually exclusive gene pairs including, but not limited to, *RB1* and *KDM6A*, *RB1* and *FGFR3*, *TP53* and *FGFR3*, *TP53* and *STAG2, TP53* and *HRAS*, and *HRAS* and *FGFR3* (Figure 1B). Mutually exclusive and cooccurring gene pairs were tested using the Fisher’s exact test (Supplemental Figure 1, D and E, Methods), which identified cooccurrence between *ERCC2*-*ERBB2*,and *ERCC2*-*SF3B1* (Supplemental Figure 1E), although there were no genes that significantly cooccurred with *ERCC2* using the DISCOVER test.

In the TCGA cohort, the median nonsynonymous mutation rate was 4 mutations per Mb, *TP53* was mutated in 46% of cases, and *ERCC2* was mutated in 9% of cases (Supplemental Figure 1B). We identified a mutually exclusive relationship between *RB1* and *FGFR3*, *TP53* and *FGFR3*, *FGFR3* and *ARID1A*, and *KMT2D* and *KDM6A*, some of which have been previously described (4) (Figure 1B).

Of the 2,012 patient-derived samples, we identified 506 *ERCC2* mutations in 477 individuals, the vast majority of which were missense variants (93%). *ERCC2* variants were highly enriched (87%) in the helicase domains (HDs) of the protein compared with the expected ratio of mutations occurring randomly along the gene (Figure 1C, χ^2^ test: *P* = 6.12 × 10^–30^). The most frequent *ERCC2* variant was N238S (Figure 1D; 14% of *ERCC2*-mutant cases); however, several other recurrent mutations were also identified (e.g. S44L, T484M, and Y24C; Figure 1D and Supplemental Figure 1F). Comprehensive copy number information and/or loss of heterozygosity (LOH) estimates were available for the WES and Indiana samples (Methods), and we found that *ERCC2* missense mutations were nearly always present without loss of the second allele (Supplemental Figure 1G): 82% of *ERCC2*-mutant cases lacked LOH versus only 5% of the cases with an LOH event detected (LOH estimates were not available for 13% of the cases). Tumor mutation burden (TMB) was calculated and harmonized across different sequencing platforms by assigning a TMB z-score to each tumor (Supplemental Figure 1H, Methods). We found that *ERCC2*-mutant cases, defined as missense or truncating (stopgain, frameshift, or nonstop) variants in the HDs of *ERCC2*, demonstrated significantly higher nonsynonymous TMB compared with WT *ERCC2* cases (defined as no mutations or mutations outside of the HDs) in all 3 cohorts (Supplemental Figure 1I; pairwise Wilcoxon rank-sum tests with Holm’s correction for multiple testing, neoadjuvant: *P* = 6.3 × 10^–5^, metastatic: *P* = 2.4 × 10^–20^, TCGA: *P* = 2.3 × 10^–9^). Finally, we performed region-specific mutational signature analysis (Methods) and found that many of the missense mutations in *ERCC2* are consistent with the mutational signatures associated with APOBEC activity (Supplemental Figure 1J). However, we also observed a number of *ERCC2* mutations due to T→C changes, including the most common variant, N238S, which is not attributable to APOBEC mutagenesis.

### CRISPR-Select identifies functionally deleterious ERCC2 helicase-domain mutations.

**Figure 2:**
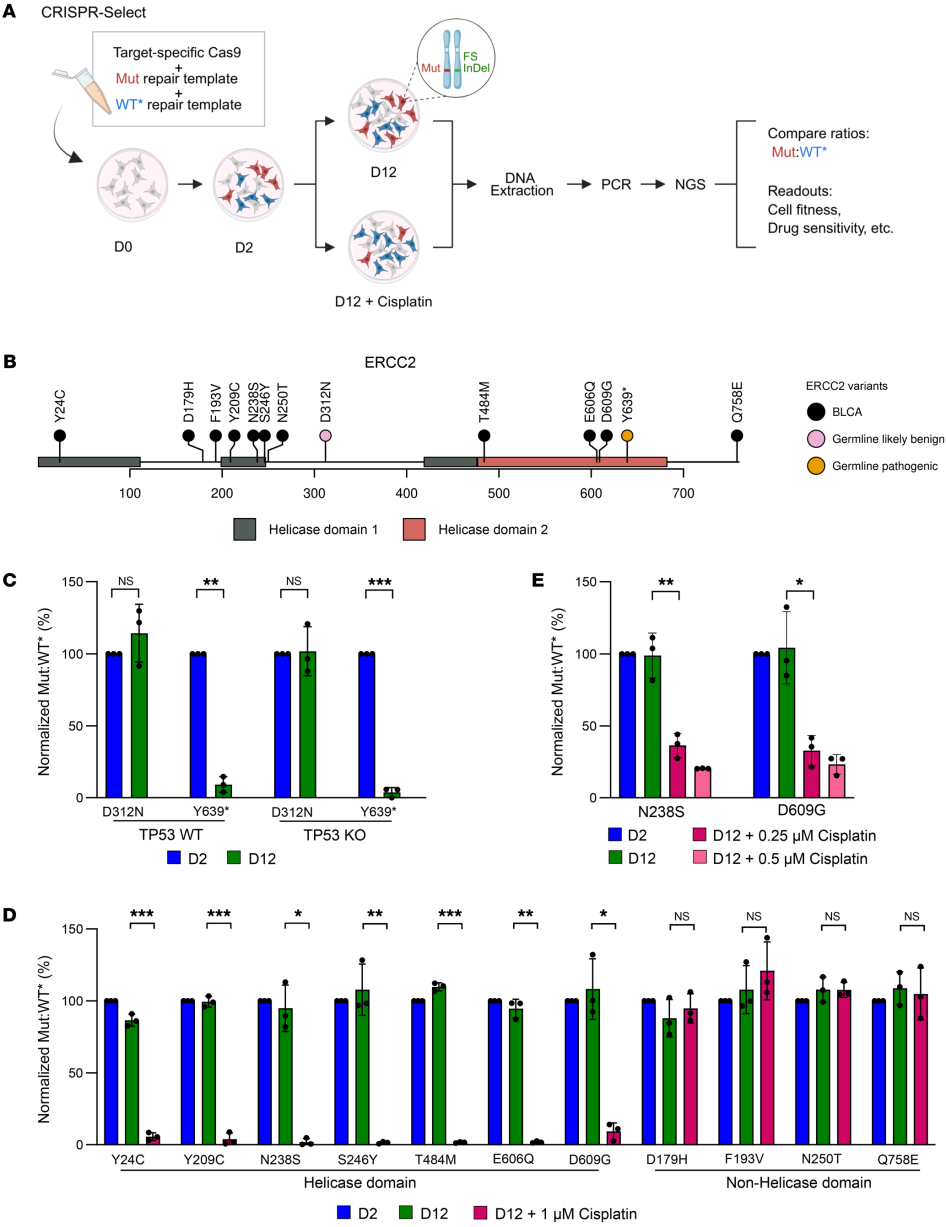
CRISPR-Select analysis establishes that helicase-domain *ERCC2* mutations confer platinum sensitivity. (**A**) CRISPR-Select workflow. iCas9-MCF10A cells are transfected with equal amount of repair templates harboring the mutation of interest (Mut) or a synonymous mutation (WT*). The WT* is used as an internal normalization control. Following CRISPR editing, most cells with a mutation of interest knocked-in on one allele will have a disruptive frameshift (fs) InDel on the other allele. Cells are harvested at day 2 (D2; initial timepoint) and the remaining cells are split into untreated or cisplatin-treated conditions and collected at D12.The region containing the Mut or WT* is deep sequenced and the Mut:WT* ratio is calculated. (**B**) Schematic representation of *ERCC2* gene structure and position of the mutations investigated by CRISPR-Select. The mutations correspond to germline mutations selected from ClinVar and somatic missense mutations identified in bladder cancer cohorts. The conserved helicase domains of *ERCC2* are depicted. (**C**) Impact of a known pathogenic (Y639*) and a likely benign (D312N) variant on cell fitness in *TP53* WT and KO iCas9-MCF10A cell lines. The normalized Mut:WT* shown corresponds to the ratio of the Mut:WT* normalized to D2. (**D** and **E**) Impact on cisplatin sensitivity of *ERCC2* variants. The normalized Mut:WT* frequencies of somatic missense mutations in (**D**) *TP53* KO iCas9-MCF10A and (**E**) bladder cancer cell lines. The error bars represent the standard deviation of 3 independent experiments. The statistical significance was determined using a paired2-tailed *t* test. *P ≤ 0.05, **P ≤ 0.01, and ***P ≤ 0.001.

*ERCC2* mutations have been associated with increased sensitivity to cisplatin-based chemotherapy in some bladder cancer cohorts, and functional analyses of selected *ERCC2* mutations have demonstrated impaired NER activity. However, the functional impact of most clinically observed *ERCC2* mutant alleles on cisplatin sensitivity has not been characterized. To quantitatively define the impact of specific *ERCC2* missense mutations on cisplatin sensitivity, we leveraged the newly developed CRISPR-Select assay (14) (Figure 2A). In this approach, CRISPR-based genome editing is used to introduce the mutation of interest (Mut) as well as a synonymous (silent) mutation (WT*) as an internal control in a single MCF10A cell population. Cell aliquots are harvested at different time intervals after editing and deep NGS is performed to monitor relative changes in mutation frequencies over time. Drug treatment was included to evaluate if the introduced *ERCC2* mutations confer increased cisplatin sensitivity. Given that *TP53* is frequently comutated with *ERCC2*, we tested the impact of *ERCC2* mutations on cisplatin sensitivity with and without cooccurring loss of *TP53*.

First, we monitored basic cell proliferation rates and did not observe any difference between *TP53* KO and *TP53* WT cell lines as measured by live microscopy (Supplemental Figure 2A). Next, we selected a known *ERCC2* pathogenic germline variant (Y639*; https://www.ncbi.nlm.nih.gov/clinvar/variation/1358482/) and a likely benign variant (D312N; https://www.ncbi.nlm.nih.gov/clinvar/variation/134117/) as controls. We introduced these alterations in the *TP53* KO and *TP53* WT cell lines and assessed the *ERCC2* Mut:WT* frequencies over time in the absence of cisplatin (Figure 2, B and C). Cells were collected on day 2 (D2, initial timepoint) and day 12 (D12) following guide RNA transfection. The Mut and WT* frequencies were calculated and then the Mut was normalized to the WT* (Mut:WT*). To compensate for experimental variability, the Mut:WT* ratio at D12 was normalized to that of D2 (Supplemental Figure 2B). The Mut:WT* frequency of Y639* decreased by approximately 80% on D12 (Figure 2C), consistent with the known impact of Y639* on *ERCC2* stability and the essentiality of *ERCC2*’s structural role as part of the TFIIH complex (21). Conversely, *ERCC2-*D312N did not affect cell fitness, supporting that this variant is benign (Figure 2C). The guide RNAs used in the CRISPR-Select experiment introduce frameshift mutations if the repair template is not used. We observed a decrease in frameshift frequency over time for guide RNAs used to edit at both Y639* and D312N positions, indicating a selection against disruptive *ERCC2* frameshift mutations for both guide RNAs, which is in line with *ERCC2*’s essential function (Supplemental Figure 2C). Together, these results support the utility of CRISPR-Select to assess the functional impact of *ERCC2* mutations.

We next investigated the impact of somatic *ERCC2* mutations identified in bladder cancer cohorts (Figure 2B) on cell fitness in the *TP53* KO (Figure 2D) and WT cell lines (Supplemental Figure 2D). In the absence of cisplatin, the variant frequency was unchanged over time for both helicase and nonhelicase mutations, suggesting that these somatic *ERCC2* mutations did not impact baseline cell fitness (Figure 2D and Supplemental Figure 2D). A decrease in guide RNA–mediated frameshift frequency over time was observed for all *ERCC2* mutations except *ERCC2*-Q758E (Supplemental Figure 2E). Q758E is in the last exon, and the guide RNA–mediated frameshift events may therefore not be as deleterious due to nonsense-mediated mRNA decay escape (22).

We next used CRISPR-Select to evaluate the impact of *ERCC2* variants on cisplatin sensitivity. We first determined the half-maximal inhibitory concentration (IC_50_) of cisplatin for *TP53*-WT and *TP53*-KO cells. Though *TP53*-WT cells were slightly more sensitive to cisplatin (IC_50_, 0.5 μM) than *TP53*-KO cells (IC_50_, 0.9 μM) (Supplemental Figure 2F), the difference was small, and we selected 1 μM cisplatin as the dose to be used for both cell lines. Two days following guide RNA transfection, an aliquot of cells was collected (D2) and the remaining cells were treated or not treated with 1 μM cisplatin and then harvested ten days later (D12). All tested helicase domain *ERCC2* mutations sensitized cells to cisplatin, as demonstrated by the statistically significant decrease in Mut:WT* frequencies in both *TP53* KO (Figure 2D) and *TP53* WT (Supplemental Figure 2D) backgrounds whereas the nonhelicase domain variants did not impact cisplatin sensitivity (Figure 2D and Supplemental Figure 2D). In a separate set of experiments in which cells were harvested on D7 and D12, no significant difference in cisplatin sensitivity was observed between *TP53*-KO and WT cells (Supplemental Figure 2G). This indicates that *TP53* loss does not influence cisplatin sensitivity induced by *ERCC2* helicase-domain mutations in vitro.

To explore the impact of *ERCC2* mutations on cisplatin sensitivity in a bladder cancer model, Cas9 and equal amounts of repair templates harboring Mut or WT* were nucleofected in J82, a malignant human urothelial cell line (23). In agreement with our prior findings, the 2 helicase domain variants, N238S and D609G, displayed increased sensitivity to cisplatin treatment (0.25 μM and 0.5 μM) but had no impact on cell fitness in the absence of cisplatin (Figure 2E). Taken together, these data demonstrate the utility of CRISPR-Select to define the functional impact of clinically observed *ERCC2* mutations on bladder cancer cell fitness and cisplatin sensitivity. Our findings show that *ERCC2* helicase-domain mutations substantially increase cisplatin sensitivity.

### Single allele editing CRISPR-Select can quantify functional impacts of heterozygous ERCC2 missense mutations.

**Figure 3:**
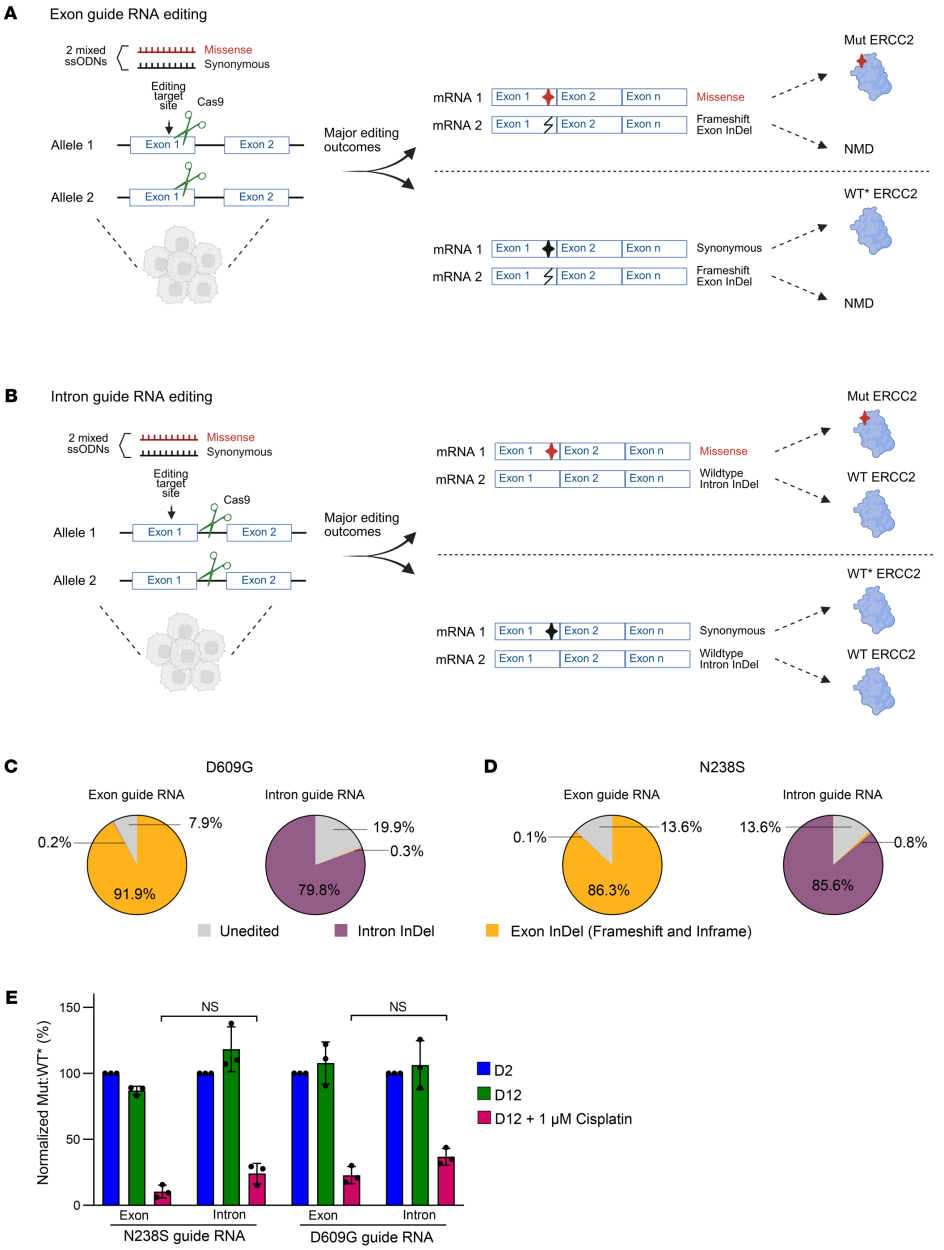
Single allele editing CRISPR-Select to quantify functional impacts of heterozygous ERCC2 missense mutations. (**A** and **B**) Principle of exon guide RNA editing compared with intron guide RNA editing. Following Cas9 cleavage, the ssODN repair templates are employed to introduce the mutation of interest (Mut) or a synonymous mutation (WT*) that are tracked by NGS. On the other allele, Cas9 introduces a cut, but, due to inefficiency of editing, this predominantly results in InDel events. Two cellular editing events resulting in Mut and WT* ERCC2 are depicted separated by dashed lines. (**A**) In exon guide RNA editing, the second allele events are frameshifts that generally are degraded by nonsense-mediated mRNA decay (NMD). (**B**) Using an intron guide RNA system, the second allele InDels are now in the noncoding region. This system can hence circumvent the formation of a high proportion of frameshifts and be used to mimic a heterozygous condition. (**C** and **D**) Quantification of exon and intron InDels at D2 in an exon guide RNA editing system compared with an intron guide RNA system. (**E**) Impact on cisplatin sensitivity of exon guide RNA and intron guide RNA for N238S and D609G variants. The normalized Mut:WT* shown corresponds to the ratio of the Mut:WT* normalized to the initial D2 timepoint. The error bars represent the standard deviation of 3 independent experiments. The statistical significance was determined using an unpaired 2-tailed *t* test.

The version of CRISPR-Select that was previously reported (14), and that we used to test the functional impact of *ERCC2* mutations in Figure 2, relies on editing of one allele to introduce the desired missense mutation coupled with highly efficient loss of heterozygosity on the second allele via InDel formation (Figure 3A). However, this genetic context differs from most bladder tumors, in which the heterozygous missense *ERCC2* mutations are present without loss of heterozygosity (LOH) of WT *ERCC2* allele(s) (Supplemental Figure 1G). To more accurately model the clinically relevant setting, we adapted the CRISPR-Select assay by using guide RNAs that target the nearest intron to the *ERCC2* mutation of interest. In this setting, the primary genome editing outcomes within a cell are as follows; (a) on one allele the donor repair templates (ssODNs) yields the desired missense (Mut) or synonymous (WT*) mutation, (b) the second allele is predominantly repaired without use of the donor repair templates, leading to intronic InDel formation that does not disrupt production of a full-length WT ERCC2 protein (Figure 3B). We term this assay “single allele editing CRISPR-Select”, as it allows introduction of heterozygous missense mutations without accompanying LOH. To validate this approach, we first compared editing outcomes using guide RNAs that targeted either the exons of *ERCC2* D609 and N238 (common sites of clinically observed mutations) or their adjacent intronic regions (in the absence of a ssODN template). As expected, the exon-targeting guide RNAs resulted in a majority of InDel events in the coding regions whereas the intron-targeting guide RNAs resulted in intronic InDel events (Figure 3, C and D). We also considered if intron guide RNA might impact regions important for RNA splicing of ERCC2. However, analysis of NGS data following editing indicated that the intron guide RNAs had a smaller effect on splicing than the exon guide RNAs (Supplemental Table 1).

We next assessed the impact of intron InDels and exon InDels on ERCC2 protein levels by transfecting cells with nontargeting, intron-targeting, or exon-targeting guide RNA only, without the addition of ssODNs, thereby inducing InDel events around the Cas9 cut site. The genomic DNA and protein were collected 3 days after guide RNA transfection. We observed an equivalent guide RNA-Cas9 efficiency (greater than 80% of modified alleles) with the intron and exon guide RNAs (Supplemental Table 2). As expected, a larger proportion of frameshift events were observed with the exon guide RNA compared to the intron guide RNA (Supplemental Table 2). Consistently, we observed a significant decrease in ERCC2 full-length protein expression with the exon guide RNA compared with the intron guide RNA (Supplemental Figure 3E). We then compared cellular fitness and cisplatin sensitivity following Mut or WT* editing with either the exon- or intron-targeting guide RNAs. Intriguingly, we observed similar cisplatin sensitivity with exon- and intron-targeting guide RNAs (Figure 3E), suggesting that helicase domain *ERCC2* mutations were sufficient to confer cisplatin sensitivity in the presence or absence of accompanying WT ERCC2 protein. More broadly, these results indicate that single allele editing of *ERCC2* mutations is feasible and support our findings obtained using the original CRISPR-Select assay (Figure 2).

### MIBC cases with ERCC2 helicase domain mutations benefit from cisplatin-based neoadjuvant chemotherapy.

**Figure 4:**
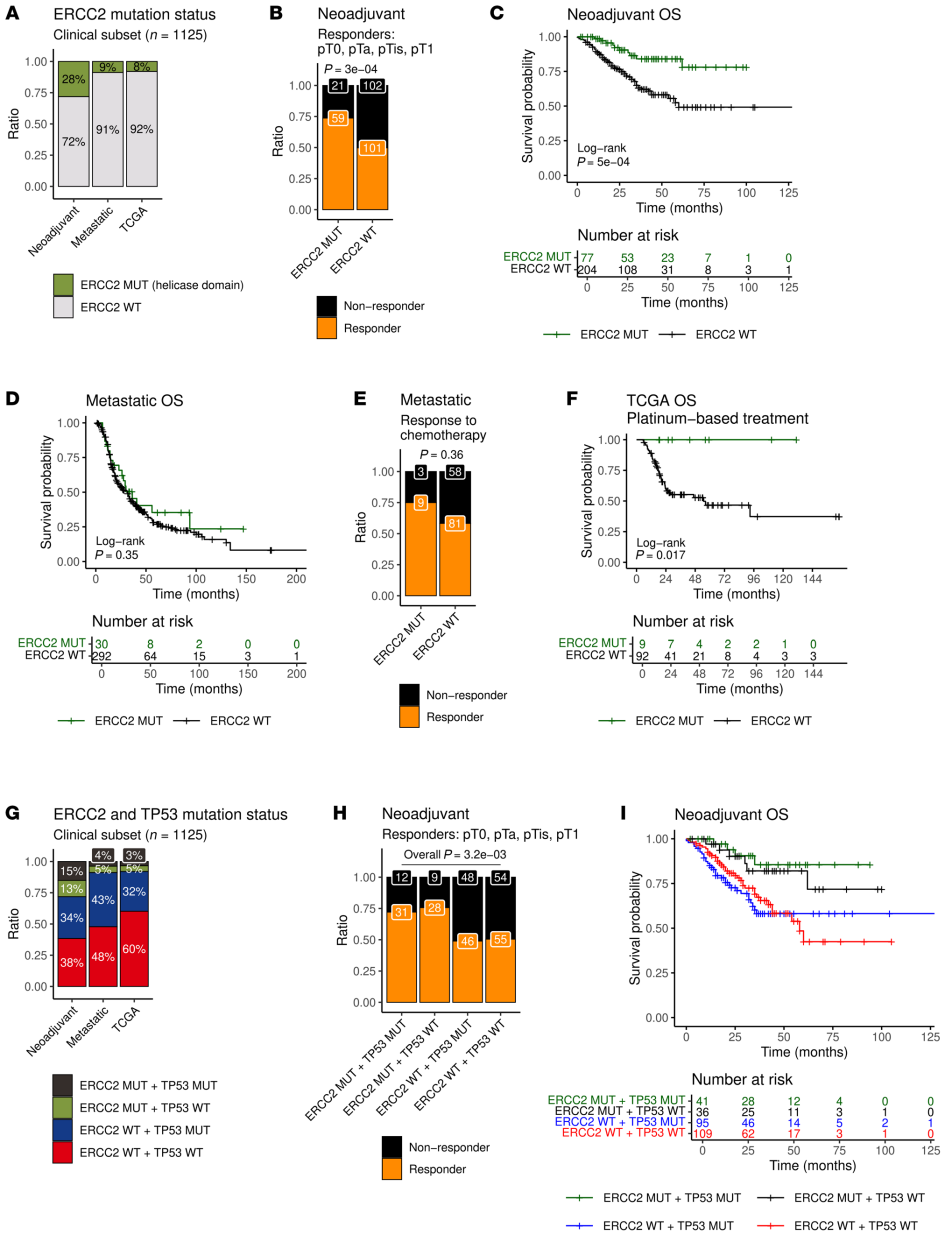
Clinical outcomes among patients in the neoadjuvant, metastatic, and TCGA cohorts. (**A**) Percentage of *ERCC2*-mutant and WT cases in the 3 cohorts (neoadjuvant, metastatic, TCGA). (**B**) Patients with helicase domain *ERCC2* mutantions were more likely to respond to cisplatin-based neoadjuvant chemotherapy (NAC). Response was defined as pT0, pTa, pTis, and pT1 (Fisher’s exact test: *P* = 3 × 10^–4^). (**C**) Kaplan-Meier plot for OS of patients in the neoadjuvant cohort stratified by *ERCC2* helicase-domain mutation status (Log-rank test: *P* = 5 × 10^–4^). (**D**) Kaplan-Meier plot for OS of patients in the metastatic cohort stratified by *ERCC2* helicase-domain mutation status (Log-rank test: *P* = 0.35). (**E**) Response to chemotherapy in the metastatic cohort (Fisher’s exact tests: *P* = 0.36). (**F**) Kaplan-Meier plots for OS of platinum-treated patients in the TCGA cohort stratified by *ERCC2* helicase-domain mutation status (Log-rank test: *P* = 0.017). (**G**) Percentage of cases grouped by *ERCC2* and *TP53* mutation status in the 3 cohorts (neoadjuvant, metastatic, TCGA). (**H**) The number of responders and nonresponders to neoadjuvant cisplatin-based chemotherapy, when response was defined as pT0, pTa, pTis, and pT1 (Fisher’s exact test: overall *P* = 0.003), among cases grouped by *ERCC2* helicase-domain mutation and *TP53* mutation status. (**I**) Kaplan-Meier plot for OS of patients in the neoadjuvant cohort stratified by *ERCC2* helicase-domain mutation and *TP53* mutation status.

To explore if *ERCC2* helicase-domain mutations may predict cisplatin response in bladder cancer, we investigated the relationship between *ERCC2* helicase-domain mutation status (Figure 4A) and patient outcomes in the assembled bladder cancer cohorts. The neoadjuvant cohort is comprised of MIBC patients who received cisplatin-based chemotherapy followed by radical cystectomy. Cisplatin responders were defined as those patients with pathologic down staging of tumors to nonmuscle invasive, node-negative disease (i.e., pT0, pTa, pTis, or pT1; and N0) at the time of cystectomy, whereas nonresponders were patients with residual muscle-invasive (pT2) or node-positive (N1) disease. Among patients with *ERCC2* helicase-domain mutations, there was a significant enrichment of responders compared with nonresponders (Figure 4B, Fisher’s exact test: *P* = 3 × 10^–4^). This enrichment persisted if a stricter definition of response (pT0, pTa, or pTis; and N0) was applied (Supplemental Figure 4A, Fisher’s exact test: *P* = 5.1 × 10^–5^). The number of cases with nonhelicase domain *ERCC2* mutations was too low to assess the association with response. Patients with helicase-domain *ERCC2*-mutant tumors had significantly longer OS compared with patients with WT *ERCC2* or a nonhelicase domain *ERCC2* mutation in our neoadjuvant cohort (Figure 4C, Log-rank test: *P* = 5 × 10^–4^).

In the metastatic cohort, there was no significant difference in OS between patients harboring a helicase-domain *ERCC2* mutation compared to patients with WT or nonhelicase domain *ERCC2* mutations (Figure 4D, Log-rank test: *P* = 0.35). For a subset of cases in the Aarhus and UC-GENOME cohorts, response to first-line chemotherapy and response to chemotherapy, respectively, were available. In the metastatic subset of the Aarhus cohort, response to first-line cisplatin-based chemotherapy was measured posttreatment by cross-sectional imaging based on the Response Evaluation Criteria in Solid Tumors (RECIST) guidelines (12). In the UC-GENOME cohort, response was reported based on investigator assessment (18). Clinical benefit was defined as any patient who had a complete response (CR), partial response (PR), or stable disease (SD). In the combined subset of Aarhus and UC-GENOME metastatic cases with available chemotherapy response data, we found no significant associations between *ERCC2* mutation status and response (Figure 4E, Fisher’s exact test: *P* = 0.36) or clinical benefit (Supplemental Figure 4B, Fisher’s exact test: *P* = 0.18), although the number of cases was limited.

In TCGA cohort, comparing OS of *ERCC2*-mutant vs WT cases, a clear separation was demonstrated when patients were stratified by receipt of platinum-based chemotherapy (Figure 4F, Log-rank test: *P* = 0.017 and Supplemental Figure 4C, Log-rank test: *P* = 0.91). Similar relationships were observed for other clinical endpoints, including progression-free interval, disease-free interval, and disease-specific survival (Supplemental Figure 4, D–I).

*TP53* is mutated in approximately 50% of all bladder cancer cases, including approximately 50% of *ERCC2*-mutant cases (Figure 4G). Notably, our CRISPR-Select analysis indicated that *TP53* status does not significantly influence the cisplatin sensitivity of *ERCC2*-mutant cells. Therefore, we investigated the impact of *TP53* mutation status on clinical outcomes following platinum-based chemotherapy in patients with versus without an *ERCC2* helicase-domain mutation. In the neoadjuvant cohort, patients with helicase-domain *ERCC2* mutations were enriched in responders regardless of *TP53* mutation status (Figure 4H, Fisher’s exact test: *P* = 3.2 × 10^–3^ and Supplemental Figure 4J, Fisher’s exact test: *P* = 8.2 × 10^–4^). We also investigated the associations of *ERCC2* and *TP53* mutation status on OS (Figure 4I, Kaplan-Meier curves) and found that helicase-domain *ERCC2* mutation status was associated with significantly longer OS (Supplemental Table 3, HR = 0.43, *P* = 0.055), but neither *TP53* status (Supplemental Table 3, HR = 1.14, *P* = 0.6) nor the interaction between *ERCC2* and *TP53* (Supplemental Table 3, HR = 0.55, *P* = 0.4) was associated with OS.

### Comparison of CRISPR-Select and computational predictions of ERCC2.

**Figure 5:**
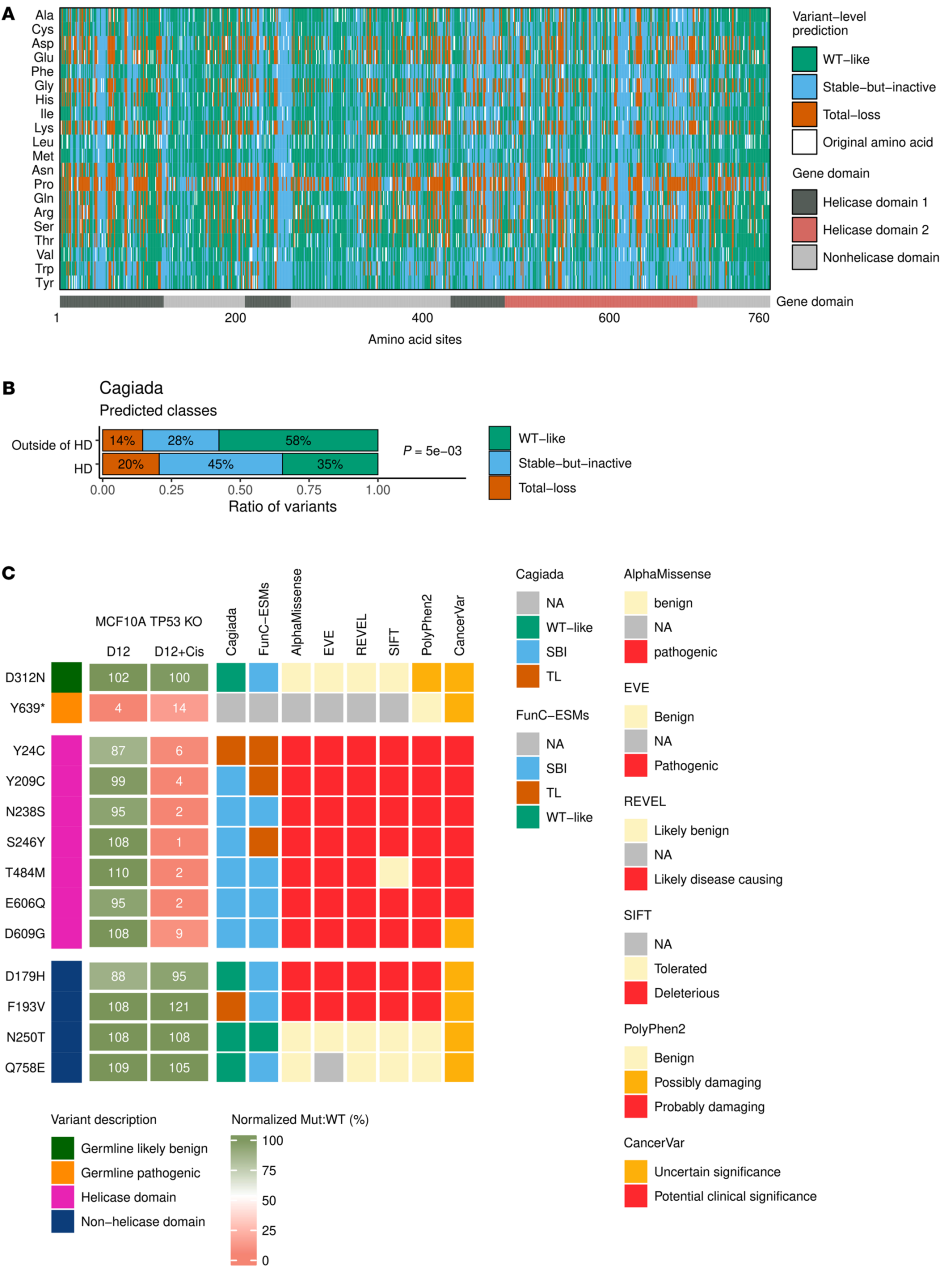
Comparison of CRISPR-Select and computational predictions of *ERCC2*. (**A**) Computational prediction of functionally important sites in *ERCC2* using the Cagiada model. The heatmap shows that 2/3 (66%) of *ERCC2* variants in the helicase domains are predicted to have a functionally or structurally detrimental effect, i.e., stable-but-inactive (SBI) (45%) or total-loss (TL) (21%) variants. (**B**) The bar plot shows the ratio of variants in each class predicted by the Cagiada model within and outside of the helicase domains (HDs) of *ERCC2*. The ratio of variants within and outside of the HDs was compared by the Fisher’s exact test: *P* = 5 × 10^–3^. (**C**) Comparison of CRISPR-Select functional experimental results using MCF10A *TP53*-KO cells and computational predictions by multiple functional and variant prediction tools. Values in “D12” and “D12+Cis” columns are showing the mean values of 3 independent experiments conducted by CRISPR-Select.

Computational models have emerged that allow fast prediction of the impact of specific mutations on certain protein functions. CRISPR-Select provides an opportunity to functionally quantify the impact of specific mutations based on endogenous locus editing. Therefore, we wished to compare the functional experimental results obtained with CRISPR-Select to various computational predictions of *ERCC2* mutation pathogenicity. To identify functionally important sites in *ERCC2*, we employed a machine learning model (24) (thereafter referred to as the Cagiada model), and a threshold-based approach called FunC-ESMs (25), or Functional Characterization via Evolutionary Scale Models. The Cagiada model classifies each variant into one of 4 categories: WT-like, stable-but-inactive (SBI), total-loss (TL), and variants with WT-like function but decreased stability. The FunC-ESMs approach is similar to the Cagiada model conceptually, although it relies on recently developed protein language models (Methods). The computational predictions of functionally important sites in *ERCC2* by the Cagiada and FunC-ESMs models are shown in Figure 5A and Supplemental Figure 5A, respectively. Both heatmaps show that the majority of variants (55% by the Cagiada model and 85% by FunC-ESMs) in *ERCC2* were predicted to be either SBI or TL variants. According to the Cagiada model, the number of variants predicted to impair protein function (SBI variants) is enriched in the HDs (45%) compared with the nonhelicase domains (28%) of *ERCC2* (Figure 5B, Fisher’s exact test: *P* = 5 × 10^–3^), which is in agreement with the expected association between functionally damaging missense variants and the HDs. However, the FunC-ESMs model did not show an enrichment of SBI variants in the HDs and noticeably appeared to overestimate the number of SBI variants in *ERCC2* (Supplemental Figure 5B, Fisher’s exact test: *P* = 0.47). In comparison with CRISPR-Select, the Cagiada model accurately predicted the effect in 10 out of 12 variants (Figure 5C). On the other hand, the FunC-ESMs method was less accurate and misclassified the control benign variant, D312N, and 3 out of 4 nonhelicase domain variants (D179H, F193V and Q758E) (Figure 5C).

In addition to the Cagiada and FunC-ESMs models, we also employed other prediction tools to assess the pathogenicity of *ERCC2* mutations including AlphaMissense (26), EVE (27), REVEL (28), SIFT (29), PolyPhen2 (30), and CancerVar (31). The predictions of *ERCC2* pathogenicity by AlphaMissense are shown in Supplemental Figure 5C. Although 68% of the total variants in *ERCC2* were predicted to be pathogenic, we observed an enrichment of pathogenic variants in the HDs compared with the nonhelicase domains of *ERCC2* (79% versus 54%, Supplemental Figure 5D, Fisher’s exact test: *P* = 8 × 10^–4^). A similarly high percentage of predicted pathogenic variants were obtained by EVE and REVEL with an enrichment of pathogenic variants in the HDs (Supplemental Figure 5, E–G).

Next, we compared CRISPR-Select findings with computational predictions of pathogenicity with a fitness-centered view (i.e., analogous to cell viability on D12 without cisplatin treatment in the CRISPR-Select assay). The majority of prediction tools characterized the benign variant (D312N) as benign, except PolyPhen2 and CancerVar, which predicted D312N as “Possibly damaging” and a variant of “Uncertain significance”, respectively (Figure 5C and Supplemental Figure 5H). For the cancer-associated helicase domain variants (Figure 5C and Supplemental Figure 5H), the computational tools predicted the variants to be pathogenic. However, CRISPR-Select did not identify a fitness impact of these variants at baseline. Rather, only in the presence of cisplatin did CRISPR-Select identify functional impacts of these helicase-domain missense variants. Finally, we also interrogated several nonhelicase domain mutations. The predicted benign impacts of N250T and Q758E by almost all tested computational methods was in agreement with the CRISPR-Select assessment (Figure 5C and Supplemental Figure 5H). However, several of these tools labeled the D179H and F193V mutations as pathogenic (Figure 5C and Supplemental Figure 5H), which contrasts with the result from CRISPR-Select that found neither a fitness impact nor cisplatin treatment impact of these variants. Thus, while computational analysis provides complementary insights to precision functional assays, caution should be taken as these methods do not necessarily account for the complex nature of the systems they address.

## Discussion

In this study, we assembled the largest cohort of *ERCC2*-mutant bladder cancer cases analyzed to date. The size of the cohort and the accompanying genomic, clinical, and novel functional data collected using CRISPR-Select has allowed us to comprehensively define frequencies and functional impacts of *ERCC2* mutations in bladder cancer.

Our cohorts consisted of distinct bladder cancer clinical states. MIBC patients present with localized (clinical T2-4 N0 M0) disease and are commonly treated in a curative-intent fashion with NAC followed by radical cystectomy (32). Recently, the addition of perioperative durvalumab (anti-PD-L1) has been shown to further improve survival (33, 34). The frequency of *ERCC2* mutations was 19% in the neoadjuvant cohort, but the actual frequency of *ERCC2* mutations in unselected patients with MIBC is likely to be somewhat lower, as our neoadjuvant cohort incorporated patients who were specifically included based on their robust response to NAC. Supporting this idea, the TCGA cohort is comprised primarily of newly diagnosed MIBC patients, and the *ERCC2* mutation frequency was 9%. The *ERCC2* mutation frequency was 11% in the metastatic cohort, the largest and most clinically heterogeneous cohort in our study. Taken together, the *ERCC2* mutation frequency in a cohort is likely to depend upon factors including clinical stage and treatment history. For example, the *ERCC2* mutation frequency in a cohort of MIBC patients treated with cisplatin-based NAC is likely to be higher than in the subset of these same patients who ultimately develop metastatic disease, since *ERCC2*-mutant patients are more likely to have a complete response — and thus less likely to develop metastatic disease — than patients lacking a tumor *ERCC2* mutation.

In addition to the frequency of *ERCC2* alterations, the association between *ERCC2* mutations and clinical outcomes also varied across disease states. There was a significant correlation between *ERCC2* mutations and improved clinical outcomes in the neoadjuvant cohort. MIBC patients in the neoadjuvant cohort were treatment-naive, and all received cisplatin-based chemotherapy. In addition, because all patients underwent radical cystectomy following neoadjuvant therapy, pathological tumor assessment could be used as a sensitive and direct surrogate of tumor cell sensitivity to cisplatin-based chemotherapy. Unlike the neoadjuvant cohort, there was no association between *ERCC2* mutation status and clinical outcomes in the metastatic cohort. Several factors could be contributing to the lack of association, including the greater clinical heterogeneity among the metastatic patient population as well as the challenge of using survival as a surrogate for cisplatin sensitivity, given that additional factors such as overall patient health and treatment-related toxicity can also impact survival in the metastatic setting. Finally, metastatic bladder cancer patients are often treated with multiple lines of therapy, and it is possible that acquired cisplatin resistance mechanisms may overcome or offset the sensitizing impact of an *ERCC2* mutation.

Functionally, *ERCC2* is a DNA helicase that couples ATP hydrolysis with DNA duplex unwinding, and nearly all observed *ERCC2* alterations were missense mutations within 1 of the 2 conserved HDs. Several mutational hotspots were observed at sites in both HDs, including N238S, T484M, S44L, and several others (Figure 1D and Supplemental Figure 1F). It is currently unknown why these specific missense mutations are more common. As discussed below, the functional impact of all tested helicase domain mutations appears to be similarly profound. It is possible that these recurrent mutations provide an as-yet uncharacterized fitness advantage; alternatively, the mutations simply arise more frequently due to a particular localizing aspect of the mutagenic process. Interestingly, we found that the most frequent missense mutations caused by C→T and C→G substitutions are consistent with APOBEC activity; however, *ERCC2* is also impacted by T→C mutations, which are not attributed to APOBEC mutagenesis (Supplemental Figure 1J).

Previous efforts from our group and others used complementation-based approaches to test the functional impact of specific *ERCC2* mutations on NER pathway activity and cisplatin sensitivity (5, 13). However, these approaches are limited by low throughput and nonphysiologic expression of the mutant alleles. CRISPR-Select was recently developed to address these and other shortcomings of traditional functional assays (14, 15). CRISPR-Select is a highly scalable NGS-based approach that overcomes many of the shortcomings of complementation-based assays and provides a quantitative and scalable approach to functional analysis of mutant alleles. CRISPR-Select is a particularly attractive approach for studying DNA repair genes, which are frequently mutated across numerous tumor types, but many of the observed mutations are of unclear functional relevance. By studying the impact of specific mutations on growth in the presence and absence of DNA damage, the impact of each mutation on tumor cell viability as well as DNA repair capacity can be quantified. In addition, the CRISPR-Select platform can be adapted to study the impact of gene alterations on specific DNA repair properties such as γ-H2AX foci formation, and others.

We leveraged CRISPR-Select to define the functional impact of clinically observed *ERCC2* mutations. Although none of the clinically observed *ERCC2* missense mutations significantly impacted cell viability in the untreated conditions, all helicase domain mutations resulted in a profound increase in cisplatin sensitivity, as evidenced by the near loss of representation of mutant-expressing cells by 7–12 days following cisplatin treatment (Figure 2D and Supplemental Figure 2, D and G). Based on these findings, it does not appear that there is a gradient effect of different *ERCC2* mutations on cisplatin sensitivity; rather, all prevalent helicase domain mutations provide a similar and profound sensitizing effect. In addition, we did not detect an impact of *TP53* status on the cisplatin-sensitizing effect of *ERCC2* helicase domain mutations, which aligns with clinical data showing no impact of *TP53* status on survival outcomes in MIBC patients treated with NAC (Figure 4I).

Our initial CRISPR-Select assay introduces the desired missense mutation by editing one allele while inducing highly efficient LOH through InDel formation in the second allele (as described in Figure 3A) (14). While this approach clearly demonstrated a cisplatin sensitizing effect of clinically observed *ERCC2* HD mutations (Figure 2), it does not fully recapitulate the genetic context of bladder cancer because most *ERCC2*-mutant bladder tumors harbor a heterozygous missense *ERCC2* mutation without accompanying LOH (Supplemental Figure 1G). To address this limitation, we modified the CRISPR-Select assay by employing an intron-targeting gRNA strategy, thereby avoiding disruptive exonic InDels (Figure 3B). We term this method single allele editing CRISPR-Select, as it creates a heterozygous missense mutation in one allele combined with an intact (WT) coding region in the second allele. We applied this approach to several *ERCC2* mutations and found that the sensitizing effect of HD mutations is similar. Thus, our novel single allele editing approach (Figure 3) validated findings from CRISPR-Select obtained with exonic gRNA (Figure 2) and suggests that *ERCC2* HD mutations may be acting via a dominant-negative mechanism. More broadly, single allele editing CRISPR-Select has the potential for numerous future applications, enabling the quantitative analysis of mutations in other genes with functional effects in the heterozygous state.

In addition to directly testing the functional impact of clinically observed *ERCC2* mutations using CRISPR-Select, we also leveraged multiple computational models to predict the pathogenicity and mechanistic consequences of *ERCC2* mutations. While these computational tools cannot incorporate explicit information about drug sensitivity and are not trained specifically for this task, they can collectively offer predictions of functional deficiency and/or instability. Generally, there was agreement among the models, with at least two-thirds of the mutations in the HDs of *ERCC2* predicted to be pathogenic or detrimental to protein function and/or structure (Figure 5, A and B, and Supplemental Figure 5, A–G). However, the comparison between CRISPR-Select experimental results and these computational outputs often yielded contradictory results (Figure 5C and Supplemental Figure 5H). We suspect that the disagreements between experimental and computationally predicted results, as well as the high ratio of predicted functionally detrimental or pathogenic variants by the computational models, may partly stem from conflated signals related to the dual roles of *ERCC2* in transcription and DNA repair, with the latter particularly key for the cellular cisplatin response. These findings highlight the importance of functional assays like CRISPR-Select to define the context-specific effects of *ERCC2* and other DNA repair gene mutations that computational predictions alone cannot achieve.

Our data demonstrate that clinically observed *ERCC2* helicase-domain missense mutations strongly sensitize bladder cancer cells to cisplatin. The potential to use mutations in *ERCC2* and other DNA repair genes to guide therapy decisions is being investigated in several clinical trials. Alliance A031701 (NCT03609216) is a Phase II clinical trial in which patients with newly-diagnosed MIBC are treated with 6 cycles of neoadjuvant gemcitabine plus cisplatin (GC). Tumor NGS is performed, and patients with a predicted deleterious alteration in *ERCC2* (or any of 8 other DNA repair genes) who experience a complete clinical response following GC are able to forego standard-of-care radical cystectomy and instead undergo close surveillance with imaging and cystoscopy. The trial is on going and has potential to provide support for biomarker-driven approaches that can maximize cure rates as well as patient quality of life. CRISPR-Select and other functional approaches can provide critical insights regarding the impact of mutations on clinically relevant properties of *ERCC2* and other DNA repair proteins, and therefore may ultimately be helpful in guiding individualized treatment approaches for patients with bladder cancer or other tumor types.

## Methods

### Sex as a biological variable

Sequencing samples and clinical information were collected from male and female patients with bladder cancer. However, sex was not considered as a biological variable.

### Cohorts and patient characteristics

In this study, 2,2012 bladder cancer cases with clinical and/or genomic information were assembled and analyzed (Supplemental Figure 1A). The complete data set consists of 675 whole-exome sequencing (WES) and 1,337 targeted panel sequencing patient-derived tumor samples collected from 8 bladder cancer cohorts.

### TCGA cohort

The TCGA cohort contains 412 muscle-invasive, high-grade urothelial tumors (T1 [*n* = 1], T2–T4a, N0–3, M0–1) analyzed by WES (4). The TCGA BLCA somatic simple nucleotide variants, allele-specific copy number segments (ASCAT, Affymetrix SNP 6.0), and clinical data were downloaded using the TCGABiolinks R package (35). Somatic simple nucleotide variants, such as single-base substitutions and insertions and deletions (indels), detected by Mutect2, were used in the downstream analyses. Genes of interest based on their genomic location were matched to allele-specific copy number segments, and if the minor copy number of the matched segments dropped to 0, then a LOH event was registered. The WES normal and tumor bam files were downloaded from the Genomic Data Commons (GDC) data portal (36) (https://portal.gdc.cancer.gov/). Allele-specific copy number profiles were also estimated by Sequenza (37) using the bam files as described previously (38). To determine the LOH status of *ERCC2* and *TP53*, the consensus between Sequenza and ASCAT was used when results from both methods were available. When ASCAT results were not available, then Sequenza was used alone.

The TCGA clinical data containing drug information was used to identify the subset of patients who received any platinum-based treatment. Clinical outcome endpoints such as OS, progression-free interval (PFI), disease-free interval (DFI), and estimated disease-specific survival (DSS) included in the TCGA Clinical Data Resource (39) were used for survival analysis.

### The DFCI-MSKCC cohort

The Dana-Farber Cancer Institute–Memorial Sloan Kettering Cancer Center (DFCI-MSKCC) cohort consists of whole-exome sequenced pretreatment tumor and germline DNA from 50 patients with muscle-invasive or locally advanced urothelial carcinoma who received cisplatin-based neoadjuvant chemotherapy (NAC) followed by cystectomy (5). Mutation data were downloaded from cBioPortal (40) database (https://www.cbioportal.org/study/summary?id=blca_dfarber_mskcc_2014). The normal and tumor bam files were downloaded from the Database of Genotypes and Phenotypes (dbGaP) upon request (https://www.ncbi.nlm.nih.gov/gap/) using the phs000771 accession code. Allele-specific copy number profiles were estimated by Sequenza (37) as described previously (38). Clinical data was provided by collaborators.

### Philadelphia cohort

The Philadelphia cohort consists of WES of prechemotherapy tumor and germline DNA from 48 patients with MIBC who received NAC followed by cystectomy (9, 16). Somatic single-nucleotide variants identified by Mutect (41) and computationally filtered from artifacts introduced by oxidative DNA damage during sample preparation (42) were provided by collaborators. The normal and tumor bam files were downloaded from the dbGaP upon request (https://www.ncbi.nlm.nih.gov/gap/) using the phs000771 accession code. Allele-specific copy number profiles were estimated by Sequenza (37) as described previously (38).

### Aarhus cohort

The Aarhus cohort includes 165 WES samples derived from patients with bladder cancer receiving chemotherapy (12). Of the 165 cases, 60 patients received NAC before cystectomy, and 105 patients received first-line chemotherapy upon detection of locally advanced or metastatic disease (98 cases received platinum-based chemotherapy). Somatic vcf files and copy number estimates described previously (12) were provided by collaborators. Genes of interest based on their genomic location were matched to allele-specific copy number segments determined by ASCAT, and if the minor copy number of the matched segments dropped to 0, then a LOH event was registered.

### MSK IMPACT cohort

The MSK IMPACT cohort consists of 329 samples derived from 288 individual patients with bladder cancer sequenced by the Memorial Sloan Kettering-Integrated Mutation Profiling of Actionable Cancer Targets (MSK IMPACT) targeted sequencing panel (43). For 286 patients, mutation data including small-scale mutations were reported in the GENIE (v16.1) public dataset (44) and were used in the downstream analysis. Of the 286 patients, 38 patients who received NAC and had available clinical information were included in the downstream analysis. The remaining 248 cases were excluded from the downstream analysis because clinical information was not available.

### Indiana cohort

The Indiana cohort contains 88 samples from patients who received NAC followed by cystectomy and had well-annotated clinical data. Tumor-only DNA-seq was performed by Myriad Genetics using the standard analysis, which is used for the commercial MyChoice testing, just on an expanded number of genes. Of the analyzed genes, we obtained information regarding *ERCC2* and *TP53* somatic mutation and LOH status. The reported mutations in *ERCC2* and *TP53* at the cDNA level were processed by TransVar (45) to identify their genomic origins using the hg38 reference genome.

### DFCI Oncopanel cohort

The DFCI Oncopanel cohort consists of 769 patients diagnosed with urothelial cancer with available targeted tumor DNA-seq performed by the OncoPanel assay (17, 46). When multiple samples were available for a given individual, then the following sample was prioritized for analysis: primary origin, more recent panel version, and higher number of detected variants. 132 cases were treated with platinum-based chemotherapy.

### The UC-GENOME cohort

The Urothelial Cancer — genomic analysis to improve patient outcomes and research (UC-GENOME) cohort includes 218 patients with metastatic urothelial cancer (18), of which primary tumors were collected for the majority of patients (87%) with the remaining samples from metastatic sites (13%). Most patients had a bladder primary tumor at initial diagnosis with high-grade and/or invasive disease (83.5%). Tumor-only targeted DNA sequencing by Caris Life Sciences was successful for 191 patients. The UC-GENOME mutation data was obtained from cBioPortal (40) database (https://www.cbioportal.org/study/summary?id=blca_bcan_hcrn_2022).

### ERCC2 and TP53 mutation status

Somatic small-scale variants detected in the samples were annotated by InterVar (47). Samples with missense or truncating (stopgain, frameshift, or nonstop) variants in the helicase domains of *ERCC2* were categorized as *ERCC2*-mutant cases (ERCC2 MUT), and patients with *ERCC2* mutations outside of the helicase domains or patients without *ERCC2* mutations were annotated as *ERCC2* WT cases.

Patients with at least a pathogenic or likely pathogenic mutation determined by InterVar in *TP53* with or without LOH of the second allele were categorized as *TP53*-mutant cases (TP53 MUT). Cases with *TP53* deep deletions were also categorized as TP53 MUT. Patients without the presence of *TP53* pathogenic or likely pathogenic mutations were categorized as *TP53* WT cases, including variants of uncertain significance.

### Mutual exclusivity and cooccurrence analysis

#### DISCOVER.

The Discrete Independence Statistic Controlling for Observations with Varying Event Rates (DISCOVER) test (19) implemented in the discover R package was used to identify mutually exclusive and cooccurring gene pairs. DISCOVER is based on a null model that takes into account the overall tumor-specific alteration rates when deciding whether alterations cooccur more or less often than expected by chance (19). Alteration matrices in all 3 cohorts (neoadjuvant, metastatic, and TCGA) were constructed from nonbenign, exonic, and splicing mutations annotated by InterVar (47) separately for mutations detected by WES and targeted panel sequencing. Pairwise testing of mutual exclusivity and cooccurrence of significantly mutated genes (4) were performed.

#### Fisher’s exact test.

Pairwise mutual exclusivity and cooccurrence between significantly mutated genes (4) were tested with the Fisher’s exact test as well using the constructed alteration matrices (Supplemental Figure 1, D and E). As described by Canisius and colleagues (19), the Fisher’s exact test is too conservative as a mutual exclusivity test and anticonservative as a cooccurrence test; however, we decided to report this analysis too. The Benjamini-Hochberg procedure was used to correct for multiple testing with a *P* < 0.01 significance level.

### Tumor mutation burden harmonization

TMB was uniformly calculated for each sample as the number of nonsynonymous mutations in coding regions per megabase (Mb) of genome covered. For WES samples, 38 Mb was used to approximate exome size as previously described (48). The DFCI Oncopanel nonsynonymous mutation counts were divided by the number of bases covered in each OncoPanel version: 0.753334 Mb (v1), 0.826167 Mb (v2), and 1.315078 Mb (v3) (49). The MSK IMPACT nonsynonymous mutation counts were divided by 0.896665, 1.016478, and 1.139322 Mb for the 341-, 410-, and 468-gene panels, respectively (49). For the MSK-IMPCT 505-gene panel, the target BED file was used to estimate the covered genome size (1.25964 Mb). For samples that were sequenced by Caris Life Sciences, TMB was calculated as the sum of nonsynonymous mutations divided by 1.4 Mb, as described previously (50). Tumor mutation burden calculated using different sequencing platforms was harmonized following the procedure developed by Vokes et al. (49) and briefly summarized in the Supplemental Methods.

### Region-specific mutational signature extraction

In order to examine whether the cancer-associated helicase-domain *ERCC2* mutations can be attributed to the mutagenic APOBEC activity, we performed a region-specific mutational signature extraction using the MutationalPatterns (51) R package. Somatic mutations mapped to the grch38 reference genome in samples with *ERCC2* helicase-domain mutations were pooled and restricted to the genomic location of *ERCC2*. The 96-channel single-base substitution mutational spectrum was determined. Fitting of previously defined signatures (COSMIC v3) (52) by a nonnegative least-squares approach was used to estimate the contribution of signatures previously found in BLCA (SBS1, SBS2, SBS5, SBS8, SBS3, and SBS40) (52).

### Survival analysis

The Kaplan-Meier method was used to estimate the survival curves with the survival and survminer R packages. The log-rank test was used to compare the survival curves. The Cox proportional hazards model was used to model the effects of *ERCC2* helicase domain mutation status, *TP53* mutation status, and their interaction on the OS of patients in the neoadjuvant cohort.

### Computational predictions of ERCC2 pathogenicity

The pathogenicity of variants in *ERCC2* was assessed by several different methods: AlphaMissense (26), EVE (27), REVEL (28), SIFT (29), PolyPhen2 (30), and CancerVar (31). Details are provided in the Supplemental Methods.

### Functional predictions of ERCC2

To identify functionally important sites in *ERCC2*, 2 methods were employed: (1) a machine learning model, referred to as the Cagiada model (24), and (2) a threshold-based approach called FunC-ESMs (Functional Characterization via Evolutionary Scale Models) (25). Details are provided in the Supplemental Methods.

### Cell culture

Immortalized human breast epithelial cells expressing doxycycline-inducible Streptococcus pyogenes Cas9 (iCas9-MCF10A) were a gift from Roderick L. Beijersbergen, The Netherlands Cancer Institute, and iCas9-MCF10A TP53 KO cell line was generated as previously described (53). MCF10A cell lines were cultured in DMEM/F-12,HEPES (Thermo Fisher Scientific, 31330038) supplemented with 5% (v/v) horse serum (Thermo Fisher Scientific, 26050088), 1% (v/v) Penicillin-Streptomycin (Gibco, 15140-122), 10 μg/mL insulin (Sigma-Aldrich, I1882), 20ng/mL epidermal growth factor (EGF) (Peprotech, AF-100-15), 0.5 μg/mL hydrocortisone (Sigma-Aldrich, H0888), and 100 ng/mL cholera toxin (Sigma-Aldrich, C8052). The human bladder cancer cell line J82 was a gift from Pr. Lars Dyrskjøt Andersen (Aarhus University). Cells were cultured in DMEM (Gibco, 31966-021) and supplemented with 10% FBS (Cytiva, SV30160.03) and 1% (v/v) Penicillin-Streptomycin (Gibco, 15140-122). All cells were cultured at 37°C in a 5% CO_2_ humidified incubator.

### Proliferation assay

iCas9-MCF10A TP53-WT and TP53-KO cells were seeded in 6-well plates in triplicates at a density of 50,000 cells/well. The day after, cells were placed in an IncuCyte S3 Live-Cell Analysis System (Sartorius, 4647) and cultured for 72 hours. Nine images per well were taken every 12 hours. Images were analyzed with the integrated IncuCyte S3 Live- Cell Analysis Software to obtain cell confluency.

### IC50 determination

iCas9-MCF10A TP53-KO and WT cells were seeded in 96-well plates at a density of 500 cells/well. Cells were treated in triplicates and treated with different concentrations of cisplatin (0,06 μM; 0,13 μM; 0,25 μM; 0,5 μM; 1 μM; 2μM; 4μM; 8μM, and 16μM) for 96 hours. Next, nuclei were stained by incubating cells with 10 μg/mL Hoechst 33342 (Thermo Fisher Scientific, H3570) for 1 hour at 37 °C. Imaging was performed in an Olympus ScanR inverted widefield microscope, and analysis was carried out using ScanR Analysis V2.8 software. IC_50_ concentration was established using nonlinear regression analysis with a sigmoidal four-parameter logistic (4PL) curve.

### CRISPR-select

CRISPR-Select cassette design and experiments were performed as previously described (14). For N238S intron guide RNA editing, an asymmetric donor with a longer homology arm on the 3’ side was designed to increase knock-in efficiency. Lists of all guide RNAs, ssODNs repair templates, and primer sequences used are given in Supplemental Tables 4–6.

### Nucleofection

For J82 bladder cancer cell lines, CRISPR-Select cassettes and Cas9 were delivered by nucleofection using the SE Cell Line 4D-Nucleofector X Kit S (Lonza, V4XC-1032). Briefly, 250 pmol of each crRNA and tracrRNA were incubated for 10 minutes at RT. Next, 62 pmol of Alt-R Streptococcus pyogenes Cas9 Nuclease V3 (IDT, 1081059) was added to the crRNA:tracrRNA complexes and incubated for 10 minutes at RT. One million cells were resuspended in 20 μL of electroporation solution and added to the mix followed by 120 pmol of each Mut and WT* ssODN. The cell suspension was transferred to a nucleocuvette and electroporated in a Lonza 4D-Nucleofector X Unit using the CM137 program.

### Guide RNA transfection and Western blot

To assess the impact of intron guide RNA and exon guide RNA on ERCC2 protein level, cells were transfected with a nontargeting, intron, or exon guide RNA. In comparison with the CRISPR-Select experiments, no ssODNs were added, thereby introducing InDels around the Cas9 cleavage site. Three days after transfection, cells were harvested for genomic DNA and protein. Genomic DNA was prepared as previously indicated for CRISPR-Select experiments. For protein analysis, cells were lysed with RIPA buffer and treated with Benzonase for 30 minutes on ice. Proteins were migrated using SDS-PAGE and then transferred on a nitrocellulose membrane and incubated overnight with ERCC2 (1:500, 10818-1-AP) or vinculin (1:10,000, V9131). Horseradish peroxidase–linked secondary antibodies were used (Vector Laboratories) and the signal visualized by chemiluminescence (Chemidoc Biorad).

### Statistics

Figures and statistical analyses were generated using GraphPad Prism Software or R (version 4.2.2 or 4.1.0). CRISPR-Select experiments were carried out in triplicates and 2-tailed *t* tests were performed. Difference between the expected and observed frequencies in categorical data was compared using a χ^2^ test. Differences between groups were analyzed using Fisher’s exact test. Survival distributions of 2 groups were compared using Log-rank tests. The Wilcoxon rank-sum test was used to compare continuous distributions between groups. *P* values of 0.05 or less were considered significant unless stated otherwise. Figure 2A and Figure 3, A and B were generated using BioRender (https://www.biorender.com/).

### Study approvals

#### MSK IMPACT cohort.

Tumor specimens and clinicopathologic information were collected from patients who consented to IRB-approved protocol no. 12-245.

#### Indiana cohort.

Patient material and clinical information were collected from patients who consented to IRB-approved protocol no. 43377386.

#### Aarhus cohort.

Informed written consent to take part in future research projects was obtained from all patients, and the specific project was approved by the National Committee on Health Research Ethics (#1706291).

#### DFCI oncopanel cohort.

Tumor specimens and clinicopathologic information were collected from patients who consented to IRB-approved protocol nos. 11-104 or 17-000.

### Data availability

The DFCI-MSKCC (5), Philadelphia (9, 16), Aarhus (12), DFCI Oncopanel (17), UC-GENOME (18) and TCGA (4) cohorts have been published previously. The Indiana and MSK IMPACT data are available upon reasonable request addressed to the corresponding authors. Computational functional prediction data generated by the Cagiada and FunC-ESMs models for this article is available on GitHub: https://github.com/KULL-Centre/_2024_borcsok_ERCC2 (Commit ID: 8168ab2.).

## Author contributions

JB, CSS, KWM, and ZS conceptualized the project. JB, DG, DDS, and CM developed the methodology. LD, GI, BJG, HZK, and MNA provided resources. JB, DG, DD, CM, NJ, and MC performed the investigation. JB, DG, DDS, and CM were responsible for visualization. CSS, ZS, KWM, and KLL supervised the project. JB, CSS, KM, ZS, DG, and DDS wrote the original draft of the manuscript. JB, CSS, KWM, ZS, DG, DDS, NJ, LD, DRS, KLL, BG, and GI edited the manuscript.

## Supplementary Material

Supplemental data

Unedited blot and gel images

Supporting data values

## Version 1 08/15/2025

Electronic publication
