# Supplementary material for: Quantitative functional profiling of ERCC2 mutations deciphers cisplatin sensitivity in bladder cancer
Source: J Clin Invest. 2025 Aug 15;135(16):e186688. doi: 10.1172/JCI186688 (PMC12352908; doi:10.1172/JCI186688)
Supplement: Supplemental data [file jci-135-186688-s234.pdf]

Supplemental Material

Quantitative functional profiling of *ERCC2* mutations deciphers cisplatin sensitivity in bladder cancer

Authors: Judit Börcsök, Diyavarshini Gopaul, Daphne Devesa-Serrano, Clémence Mooser, Nicolas Jonsson, Matteo Cagiada, Dag R. Stormoen, Maya N. Ataya, Brendan J. Guercio, Hristos Z. Kaimakliotis, Gopa Iyer, Kresten Lindorff-Larsen, Lars Dyrskjøt, Kent W. Mouw, Zoltan Szallasi, Claus S. Sørensen

Supplemental Methods

Tumor mutation burden harmonization

As described in the Methods section, tumor mutation burden calculated using from mutation data detected by different sequencing platforms was harmonized following the procedure developed by Vokes et al. (1). Briefly, power transformations were used to normalize cohort-specific TMB distributions; Tukey's ladder of powers in the rcompanion R package was used to identify the optimal transformation coefficient. The normalized distributions were then standardized into z-scores by subtracting the transformed distribution mean and dividing by the standard deviation. Normalization and standardization of TMB distributions bring the WES and targeted panel sequencing cohort distributions into alignment (Supplemental Figure 1H). Each original cohort was normalized and standardized individually; however, the kernel density plot of unadjusted TMB values and the density plot of transformed TMB z-scores are shown for the three combined cohorts (neoadjuvant, metastatic, TCGA; Supplemental Figure 1H). The Indiana cohort was excluded from the TMB calculation and harmonization process because we only had information regarding somatic *ERCC2* and *TP53* mutations. Following TMB harmonization, TMB z-scores of *ERCC2*-mutant and WT cases were compared (Supplemental Figure 1I).

Computational predictions of *ERCC2* pathogenicity

Pathogenicity scores estimated by AlphaMissense, EVE, and REVEL, were downloaded from https://console.cloud.google.com/storage/browser/dm_alphamissense, https://evemodel.org/, and https://sites.google.com/site/revelgenomics/, respectively. EVE score and classification of the 75% most confident class assignments over all possible amino acid substitutions were used. Variants with REVEL score between 0 and 0.5 were classified as benign, whereas variants with a score ≥ 0.5 were classified as disease causing. CancerVar predictions were obtained by using the command-line python code. SIFT and PolyPhen2 scores were derived from the ANNOVAR (2) annotation software, and the following cut-off values were used to classify variants: < 0.05 deleterious and ≥ 0.05 tolerated for SIFT

score; and ≤ 0.446 benign, > 0.446 and ≤ 0.908 possibly damaging, and > 0.908 probably damaging for PolyPhen2.

Functional predictions of *ERCC2*

To identify functionally important sites in *ERCC2*, two methods were employed: (i) a machine learning model, referred to as the Cagiada model (3), and (ii) a threshold-based approach called FunC-ESMs (Functional Characterisation via Evolutionary Scale Models) (4). The Cagiada model combines statistical models for protein sequences with biophysical models of stability in a machine learning model that was trained using multiplexed experimental data on variant effects. This method classifies each variant into one of four categories: (i) wild-type-like (WT-like), with predicted wild-type-like protein function and stability; (ii) stable-but-inactive (SBI), with predicted decreased protein function but wild-type-like stability; (iii) total-loss (TL), with predicted decreased protein function and stability; and (iv) variants with wild-type-like function, but decreased stability. The FunC-ESMs model leverages pre-trained protein language models such as ESM-1b for protein "conservation" (5) and ESM-IF (Inverse Folding) to assess the impact of variants on protein stability (6). The model classifies each variant into one of three categories: (i) WT-like, (ii) SBI, and (iii) TL.

CRISPR-Select

Design

CRISPR-Select cassette design and experiments were performed as previously described (7). Guide RNAs were designed using the online software Benchling (https://benchling.com) such that the Cas9 cut site was less than 10 nucleotides away from the mutation. To prevent potential off targets for mutations farther away, an additional synonymous mutation was included in the PAM or seed region to destroy the Cas9 target site. Guide RNAs were purchased as CRISPR RNA (crRNA) and trans-activating crRNA (tracrRNA) from Integrated DNA Technologies (IDT). CRISPR-Select repair templates were designed as single-stranded oligodeoxynucleotides (ssODNs) with 45 nucleotides homology arms flanking the codon to be mutated. A synonymous mutation (WT*) was introduced in the same codon

as the variant of interest (Mut). ssODN repair templates were purchased as UltramerTM DNA Oligonucleotides from IDT.

Transfection

iCas9-MCF10A cells were transfected at 50-70% confluency. Cas9 expression was induced 24 hours prior to transfection by adding 1µg/ml doxycycline to the culture medium. For a 9.6cm^2 well, 60 pmol of each crRNA and tracrRNA in 6 µL were incubated for 10 min at room temperature (RT) for complex formation. Next, 125µl Opti-MEM Reduced Serum Medium (ThermoFisher Scientific, 31985062) was added to the mix, followed by 8 pmol of each of the Mut and WT* ssODN. To this mix, 6 µL of LipofectamineTM RNAiMAX Transfection Reagent (ThermoFisher Scientific, 13778500) and 125 µL Opti-MEM Reduced Serum Medium were incubated for 10min at RT. The mix was then dropped onto iCas9-MCF10A cells in a fresh medium containing 1µg/mL of doxycycline.

Analysis

Two days after transfection, an aliquot of cells was collected for the initial time point. The remaining cells were split into untreated and cisplatin-treated conditions (1 µM cisplatin for iCas9-MCF10A and 0.5 µM or 0.25 µM cisplatin for J82). Culture media was changed every 3 days, and cells were split when confluent. Cells were harvested at D7 or D12 after transfection for the Mut:WT* analysis. Genomic DNA was extracted, and a first round of PCR was performed to amplify the region containing the mutation. The PCR product was then used as the template for a second PCR round, in which the primers contained overhangs with sample-specific barcodes, as well as adaptors for next-generation sequencing (NGS). Amplicon library was prepared using a MiSeq Reagent Kit v2 (Illumina, MS-102-2002) and sequenced in a MiSeq instrument (Illumina, SY-410-1003), according to the manufacturer's instructions. NGS data were analyzed using the CRISPResso2 online tool (https://crispresso.pinellolab.partners.org/submission). Statistical analysis was performed with GraphPad Prism Software.

Supplemental Figures

Supplemental Figure 1

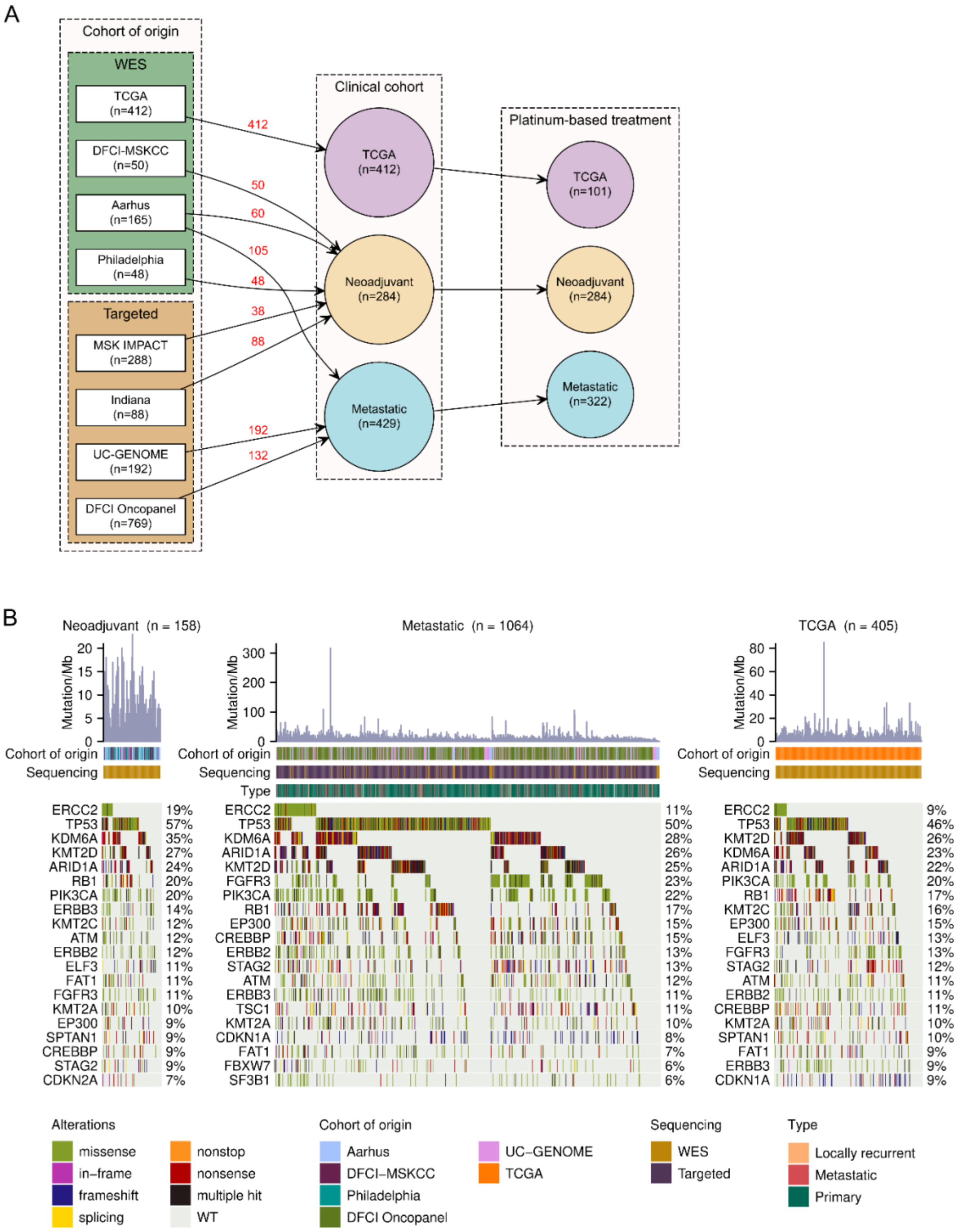

Supplemental Figure 1

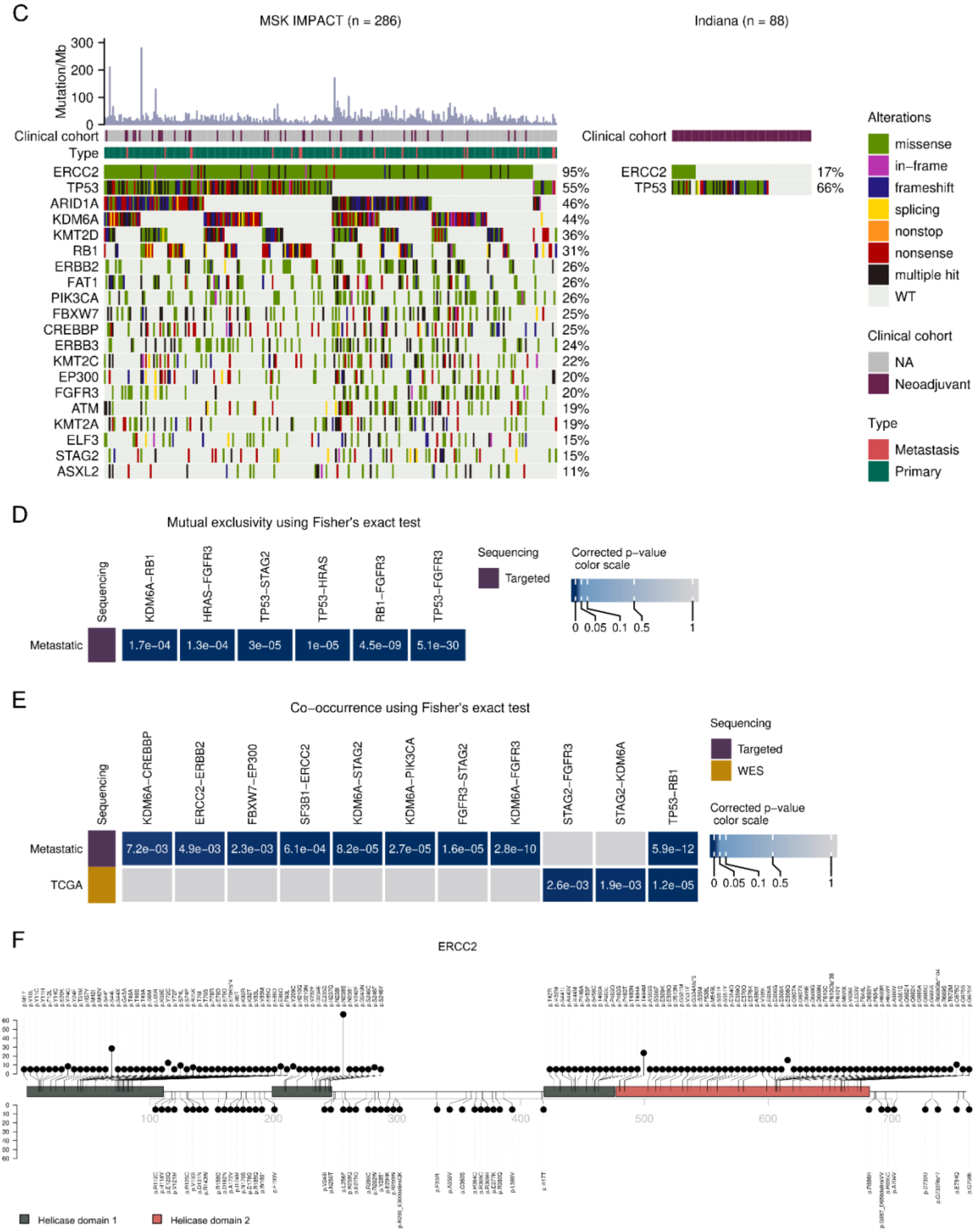

Supplemental Figure 1

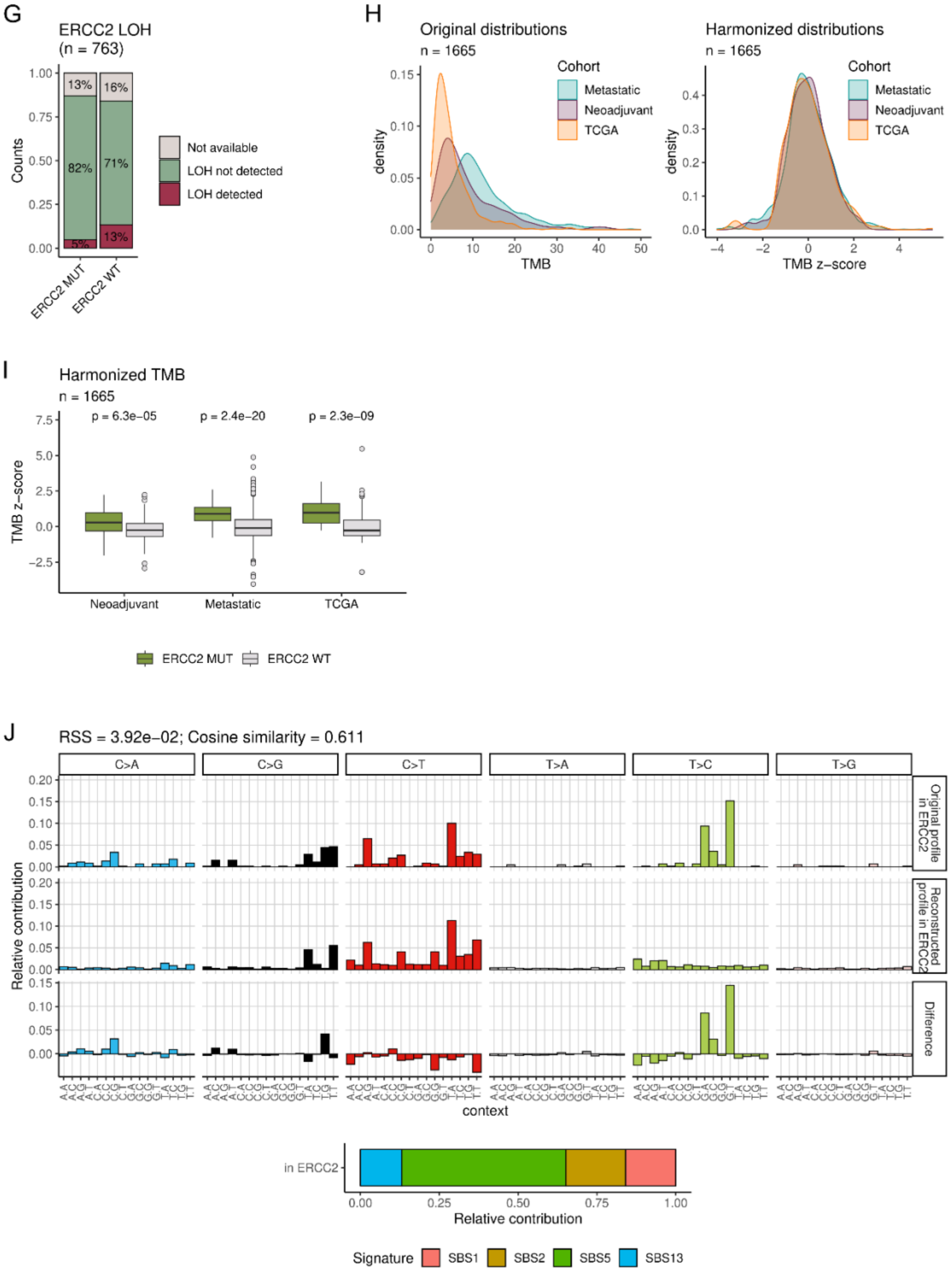

Supplemental Figure 1: A. Flowchart showing the collected cohorts and how the samples were grouped. **B.** Mutation landscape of the neoadjuvant, metastatic and TCGA cohorts. The oncoprint showing the neoadjuvant cohort does not include the MSK IMPACT and Indiana cohorts, because the MSK IMPACT cohort only contains *ERCC2*-mutant cases, and the Indiana cohort only contains mutations in *ERCC2* and *TP53*. **C.** Oncoprints for the MSK IMPACT and Indiana cohorts. **D.** Mutually exclusive gene pairs using the Fisher's exact test (Benjamini-Hochberg method to correct for multiple comparisons, $p < 0.01$). **E.** Co-occurring gene pairs using the Fisher's exact test (Benjamini-Hochberg method to correct for multiple comparisons, $p < 0.01$). **F.** *ERCC2* variants identified from the collected cohorts are mapped to the linear structure of the gene. **G.** Percentage of loss-of-heterozygosity (LOH) in available cases grouped by their *ERCC2* mutation status. **H.** Distribution of tumor mutation burden (TMB) scores before and after of the harmonization procedure. **I.** Comparison of harmonized TMB z-scores between *ERCC2*-mutant and WT cases in the neoadjuvant, metastatic and TCGA cohorts (pairwise Wilcoxon rank-sum tests with Holm's correction for multiple testing, neoadjuvant: p-value = $6.3 \cdot 10^{-5}$, metastatic: p-vale = $2.4 \cdot 10^{-20}$, TCGA: p-value = $2.3 \cdot 10^{-9}$). For each boxplot, the dark horizontal line within each box represents the median, the edges of the box represent the lower and upper bounds of the interquartile range (IQR), the upper whisker is the min(max(x), Q3+1.5·IQR) and the lower whisker is the max(min(x), Q1-1.5·IQR). **J.** Region-specific mutational signature analysis in the genomic region of *ERCC2*. The original 96-channel mutational spectrum is shown in the top plot, the reconstructed 96-channel mutational spectrum is shown in the middle plot, and the difference between the original and the reconstructed profiles is shown in the bottom plot. The relative contribution of signatures determined by fitting to the previously defined signatures that were found in BLCA is shown in the bar plot. RSS: residual sum of squares.

Supplemental Figure 2

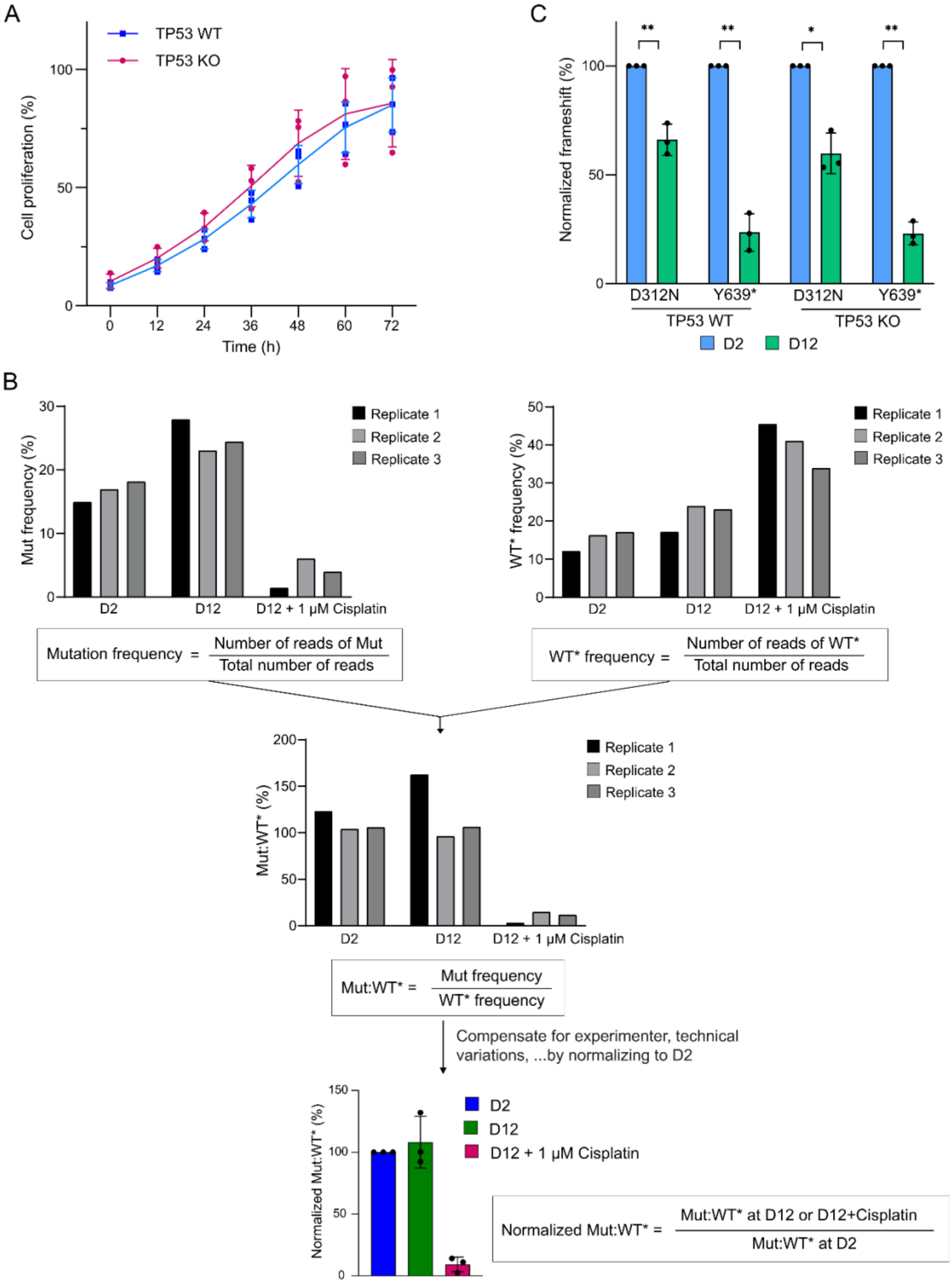

Supplemental Figure 2

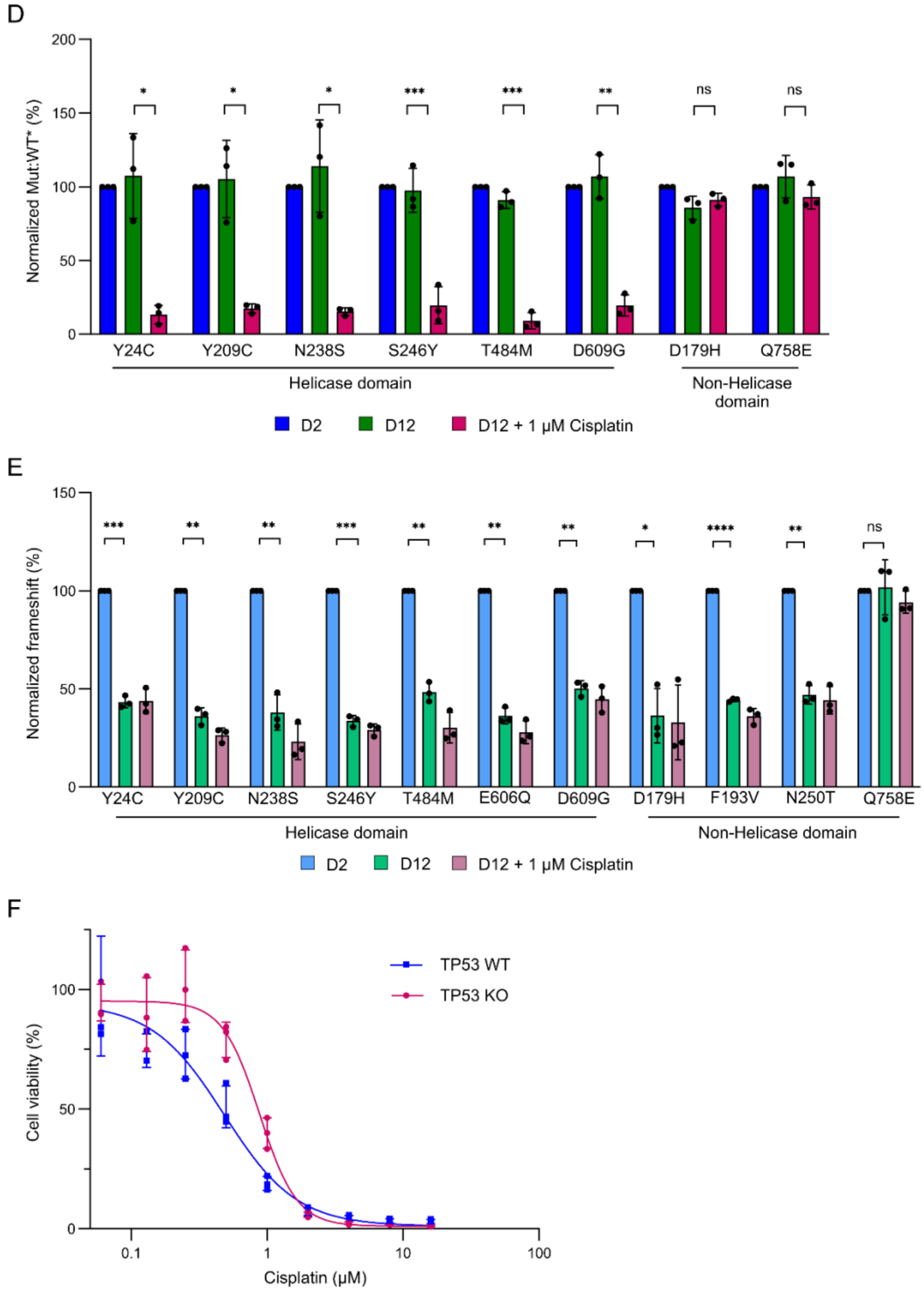

Supplemental Figure 2

G

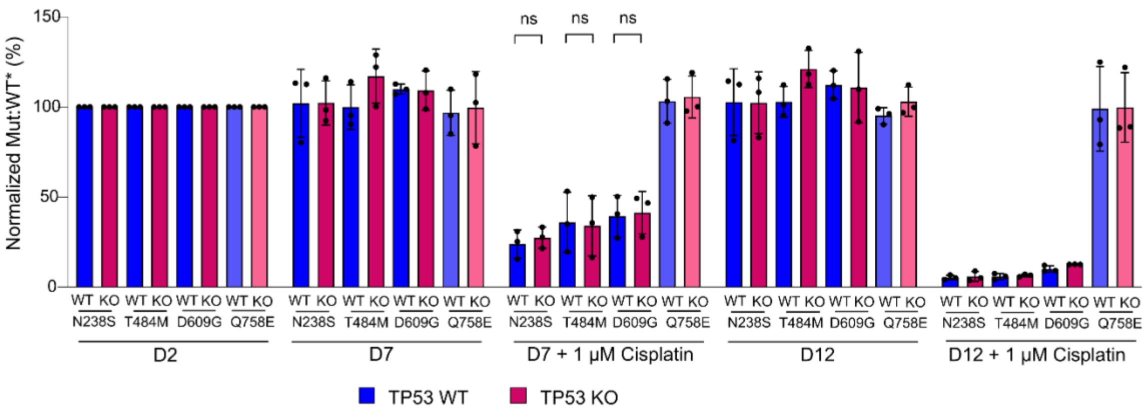

Supplemental Figure 2: A. The proliferation rate of iCas9-MCF10A *TP53* WT and *TP53* KO cells was assessed over 72h by live cell imaging. The error bars represent the standard deviation of three independent experiments. **B.** CRISPR-Select data analysis. The mutation of interest (Mut) and synonymous mutation (WT*) frequencies are first calculated for three independent triplicates. The Mut frequency is then normalized to the WT*. The Mut:WT* ratio at D12 or D12+Cisplatin is then normalized to the initial timepoint D2 to compensate for any experimental variability. **C-E.** The cisplatin sensitivity of *ERCC2* variants was investigated by CRISPR-Select in *TP53* WT and KO iCas9-MCF10A cells. **C.** The normalized frameshift frequencies of D312N (likely benign) and Y639* (pathogenic) variants. The frameshift frequency is normalized to the initial time point D2. **D.** Mut:WT* ratio of cancer-associated *ERCC2* missense mutations in *TP53* WT iCas9-MCF10A cell line, normalized to the initial time point D2. **E.** Frameshift frequencies of cancer-associated *ERCC2* missense variants in *TP53* KO iCas9-MCF10A cell line, normalized to the initial timepoint D2. **F.** iCas9-MCF10A *TP53* WT and *TP53* KO cells were treated with different concentrations of cisplatin for 96h, and cell survival was scored. The dose-response curve was used to determine half maximal inhibitory concentration (IC50) of cisplatin. The error bars represent the standard deviation of three technical replicates. **G.** The normalized Mut:WT* ratio was assessed in *TP53* WT and KO iCas9-MCF10A cells to determine whether *TP53* had an impact on cell sensitivity to cisplatin. The error bars represent the standard deviation of three independent experiments. The statistical significance was determined using a paired t-test. P-value > 0.05 (ns), ≤ 0.05 (*), ≤ 0.01 (**) and ≤ 0.001 (***).

Supplemental Figure 3

A D609G: Exon guide RNA

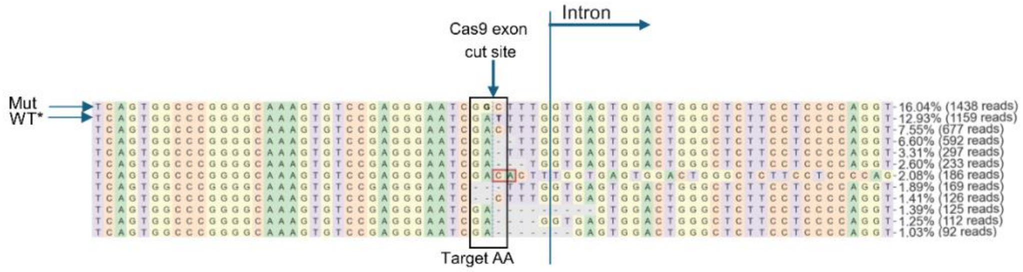

B D609G: Intron guide RNA

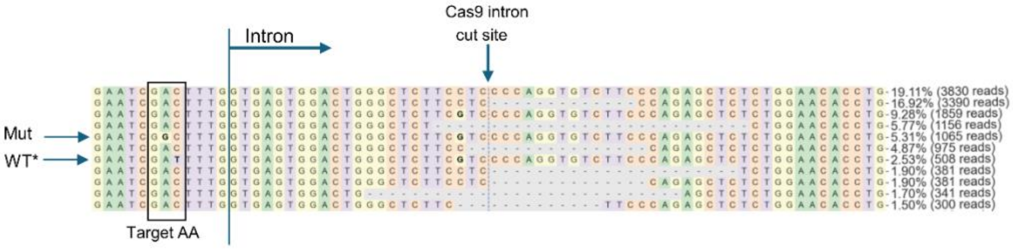

C N238S: Exon guide RNA

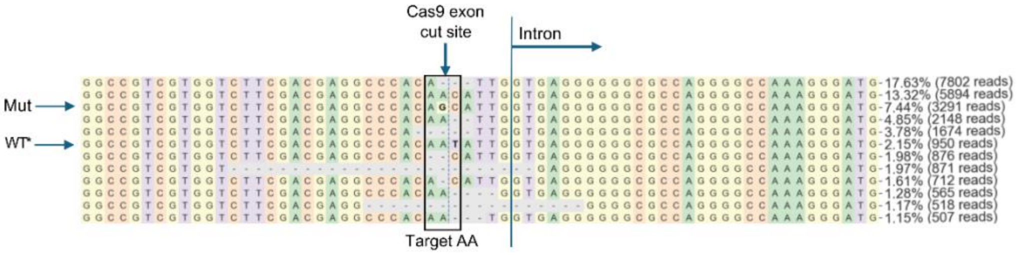

D N238S: Intron guide RNA

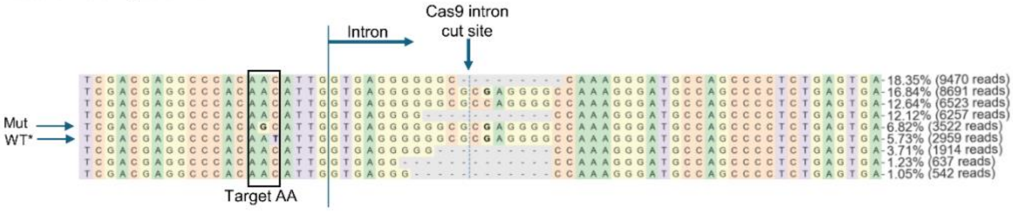

E

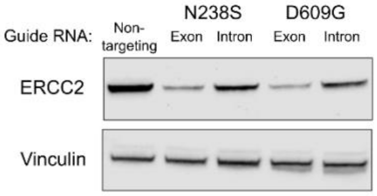

Supplemental Figure 3: A-D. Example of Mut and WT* knock-in efficiencies for D609G and N238S variants. Knock-in efficiency, higher than 1% of exon and intron guide RNAs are depicted for D609G (**A** and **B**, respectively) and for N238S (**C** and **D**, respectively). Encased in a black box are the trinucleotides corresponding to the amino acid (AA) that has been changed. Cas9 cut sites and the intron start are indicated. Depending on the guide RNA used, Cas9 cleavage is directed either to the exonic region (exon guide RNA) or intronic region (intron guide RNA). Substitutions are indicated in bold. Nucleotides in a red box indicate insertions. The dashed lines indicate deletions. **E.** Comparison of ERCC2 protein levels by Western blot following different guide RNA transfections.

Supplemental Figure 4

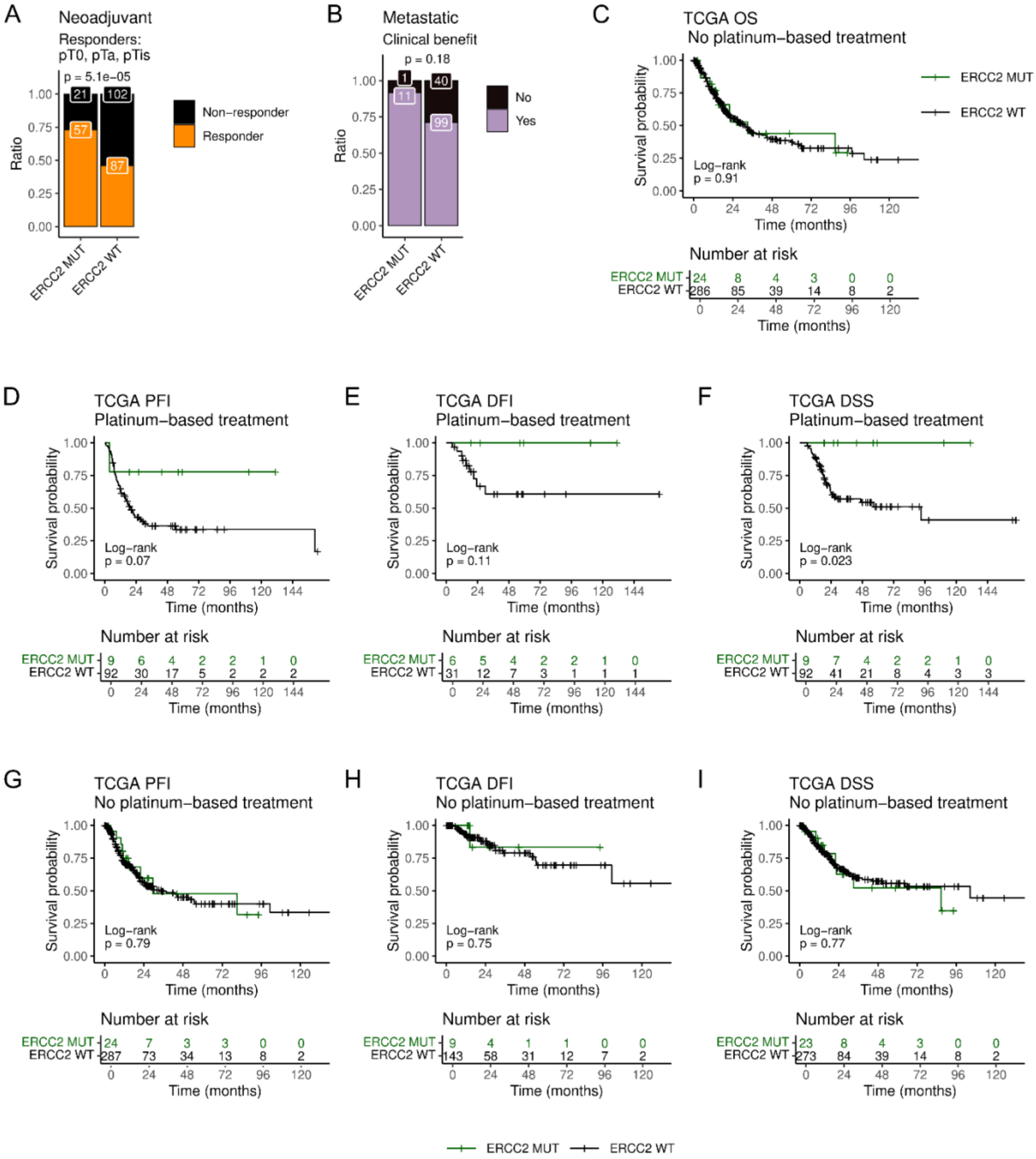

Supplemental Figure 4

J

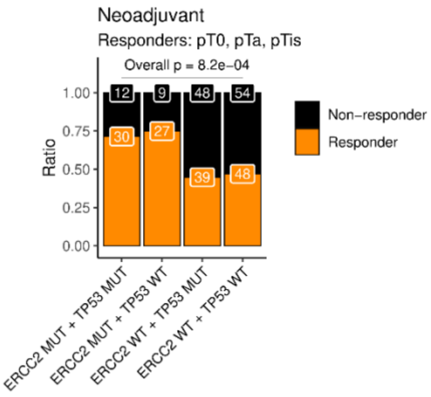

Supplemental Figure 4: A. Enrichment of responders to cisplatin-based neoadjuvant chemotherapy (NAC) among *ERCC2*-mutant cases compared with *ERCC2* WT cases in the neoadjuvant cohort when response was defined as pT0, pTa, and pTis (Fisher's exact test: p-value = $5.1 \cdot 10^{-5}$). **B.** Clinical benefit from chemotherapy in *ERCC2*-mutant vs WT cases in the subset of the metastatic cohort with available information (Fisher's exact test: p-value = 0.18). **C.** Kaplan-Meier plot of overall survival (OS) of patients not receiving platinum-based treatment in the TCGA cohort stratified by *ERCC2* helicase-domain mutation status (Log-rank test: p-value = 0.91). **D-F.** Kaplan-Meier plots of progression-free interval (PFI), disease-free interval (DFI), and disease-specific survival (DSS) of platinum-treated patients stratified by *ERCC2* helicase-domain mutation status in the TCGA cohort. **G-I.** Kaplan-Meier plots of PFI, DFI, and DSS of patients not receiving platinum-based treatment stratified by *ERCC2* helicase-domain mutation status in the TCGA cohort. P-values were calculated using the log-rank test. **J.** The number of responders and non-responders to cisplatin-based NAC when response was defined as pT0, pTa, and pTis (Fisher's exact test: overall p-value = $8.2 \cdot 10^{-4}$).

Supplemental Figure 5

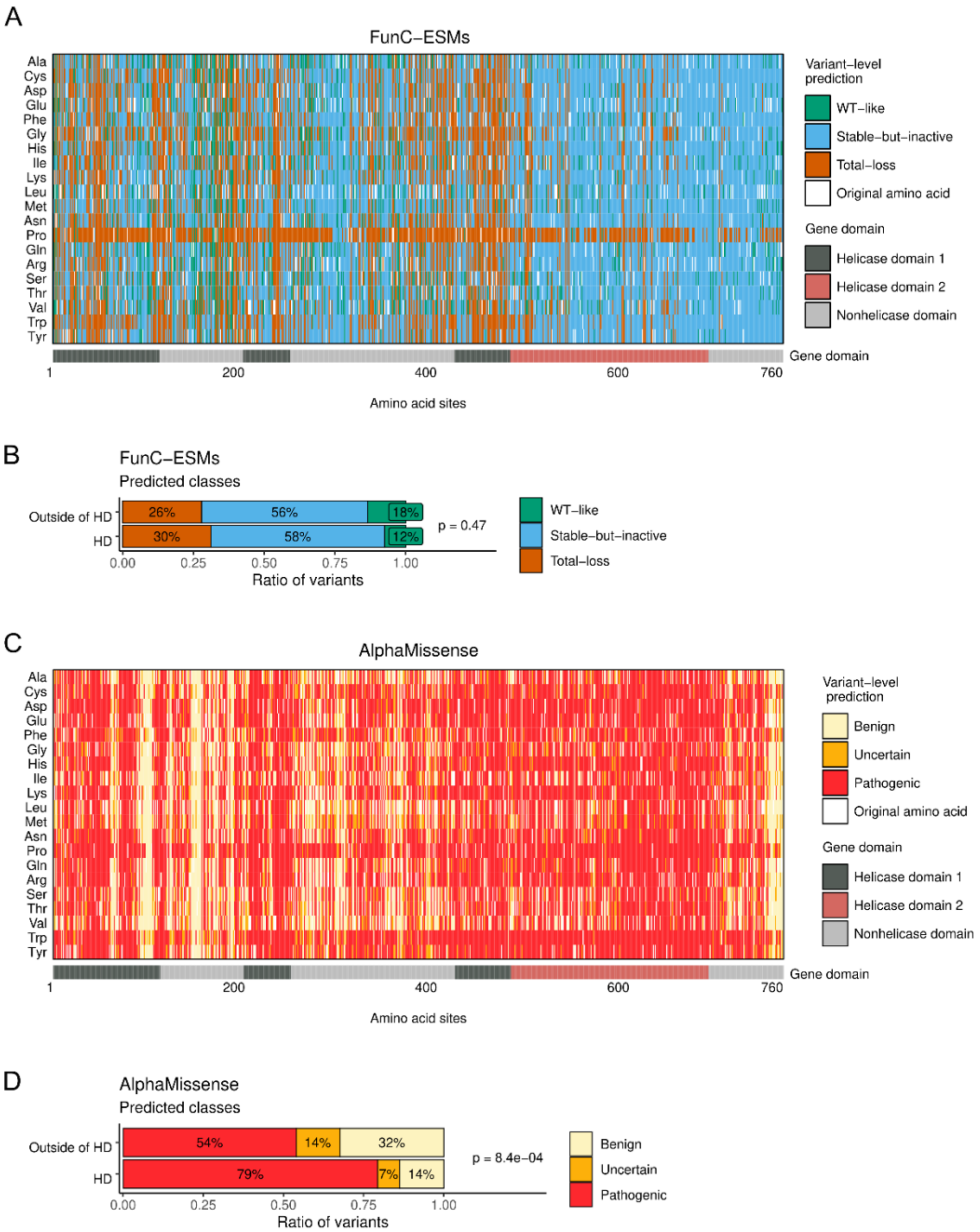

Supplemental Figure 5

E

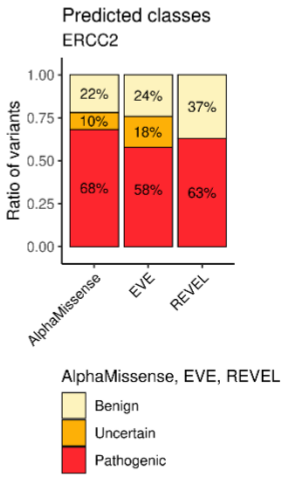

F

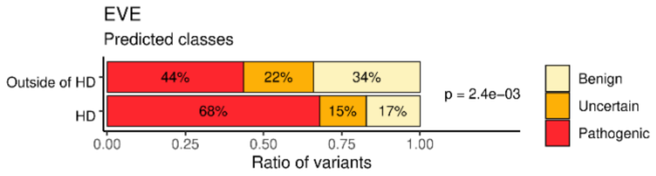

G

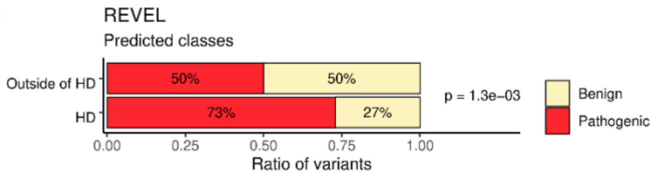

H

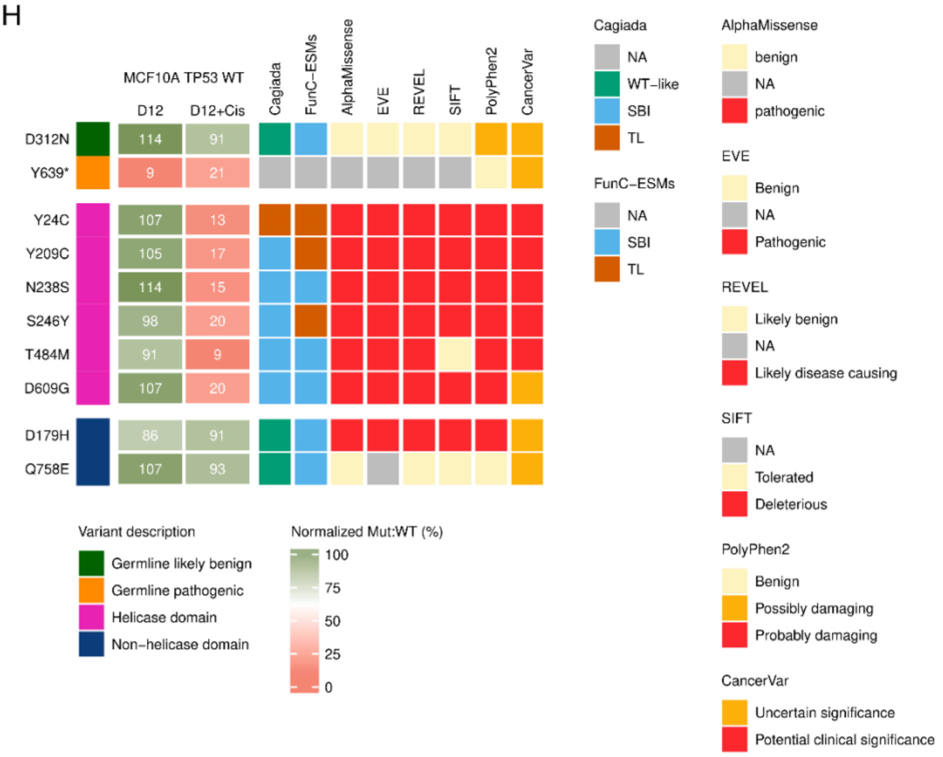

Supplemental Figure 5: A. Computational prediction of functionally important sites in *ERCC2* using the FunC-ESMs model. The heatmap demonstrates that the model apparently overestimates the number of stable-but-inactive variants. **B.** The bar plot shows the ratio of variants in each class assessed by the FunC-ESMs model within and outside of the helicase domains (HDs) of *ERCC2*. The ration of variants within and outside of the HDs was compared with the Fisher's exact test and no significant difference was observed (p-value = 0.47). **C.** A heatmap showing AlphaMissense predictions of *ERCC2* pathogenicity. **D.** The bar plot shows the ratio of variants in different classes determined by AlphaMissense within and outside of the HDs of *ERCC2*. There was a significant enrichment of predicted pathogenic variants in the HDs compared to the non-helicase domains (Fisher's exact test: p-value = $8.4 \cdot 10^{-4}$). **E.** Comparison of the ratio of *ERCC2* variants in each class predicted by AlphaMissense, EVE and REVEL. **F-G.** The ratio of *ERCC2* variants in each class within and outside of the HDs assessed by EVE and REVEL, respectively. There was an enrichment of predicted pathogenic variants in the HDs of *ERCC2* by both methods (Fisher's exact tests, EVE: p-value = $2.4 \cdot 10^{-3}$, REVEL: $1.3 \cdot 10^{-3}$). **H.** Comparison of CRISPR-Select functional experimental results using MCF10A *TP53* WT cells and computational predictions by multiple functional and variant prediction tools. Values in "D12" and "D12+Cis" columns are showing the mean values of three independent experiments conducted by CRISPR-Select.

Supplemental Tables

Supplemental Table 1: Impact on potential splice sites of exon and intron guide RNA editing for two helicase domain variants, N238S and D609G.

Variant of interest	Type of guide RNA	Unmodified splice sites	Predicted impact on splice sites
D609G	Exon	83%	17%
	Intron	99.6%	0.4%
N238S	Exon	84%	16%
	Intron	96%	4%

Supplemental Table 2: Percentage of modified alleles and frameshifts in cells transfected with non-targeting, exon, or intron guide RNA without the ssODNs, assessed by NGS sequencing of PCR amplicons corresponding to the D609 or N238 regions.

Variant of interest	Type of guide RNA	Modified alleles	Frameshift frequency
D609G	Non-targeting	1.2%	0.22%
	Exon	83.8%	60.3%
	Intron	83.9%	0.29%
N238S	Non-targeting	1.4%	0.33%
	Exon	87.6%	46.2%
	Intron	85.8%	4.4%

Supplemental Table 3: The estimated hazard ratios (HR) with upper and lower 95% confidence intervals (CIs), and associated p-values using the Cox proportional hazards model.

**Variable**		**HR**	**95% CI**	**p-value**
ERCC2 status	WT	–	–	
	MUT	0.43	0.18, 1.02	0.055
TP53 status	WT	–	–	
	MUT	1.14	0.71, 1.85	0.6
ERCC2 and TP53 status	MUT * MUT	0.55	0.14, 2.13	0.4

Supplemental Table 4: List of crRNAs' sequences.

Target mutation	20nt guide sequence (5' to 3')
D179H	CTACAACCTGGATGACCTGA
D312N	CTGCCCGACGAAGTGCTGCA
D609G (Exon guide RNA)	GTGTCCGAGGGAATCGACTT
E606Q	GTGTCCGAGGGAATCGACTT
N238S (Exon guide RNA)	TTCGACGAGGCCCACAACAT
Q758E	GATTGCTCAGCAGCTCTGAG
S246Y	GCGGGTGAGGTTGACGCTCA
T484M	GGCAACCTTCACCATGACGC
Y209C	TTATAGCTACCACTACCTCC
Y24C	CCGAGCAGTTCTCCTACATG
Y639*	AGGCGCGGCTGGAATACCTG
F193V	AGTATCGAGCAAGGAAGTAT
N250T	TGAGCGTCAACCTCACCCGC
N238S (Intron guide RNA)	CATTGGTGAGGGGGGCGCCA
D609G (Intron guide RNA)	CTCTGGGAAGACACCTGGGG

Supplemental Table 5: List of ssODN repair templates. The sequences of the reference (WT), the mutation of interest (MUT) and the synonymous mutation (WT*) analyzed by CRISPR-Select. Highlighted in yellow is the modified codon, with the mutation indicated in red. The PAM sequence is indicated in bold, and the vertical line "|" indicates the spCas9 cut site. When the PAM sequence is placed 10 or more nucleotides away from the mutation site, an additional synonymous mutation (highlighted in gray) is inserted between the codon to be mutated and the PAM sequence to avoid re-cutting.

**Name**	**ssODN sequence (5' to 3')**
D179H-WT	CATGGGCGTGAGGTGCCCCTCCCCGCTGGCATCTACAACCTGGATGACC|TGA**AGG**CCCTGGGGCGGC GCCAGGGCTGGTGCCCATACTTCCTT
D179H-MUT	CATGGGCGTGAGGTGCCCCTCCCCGCTGGCATCTACAACCTGGATCACC|TGA**AGG**CCCTGGGGCGGC GCCAGGGCTGGTGCCCATACTTCCTT
D179H-WT*	CATGGGCGTGAGGTGCCCCTCCCCGCTGGCATCTACAACCTGGATGATC|TGA**AGG**CCCTGGGGCGGC GCCAGGGCTGGTGCCCATACTTCCTT
D312N-WT	GCCGCCCGGGAGACGGACGCCCACCTGGCCAACCCCGTGCTGCCCGACGAAGTGCT|GCA**GGG**TG AGccccgaccccccgctgccccccagtcc
D312N-MUT	GCCGCCCGGGAGACGGACGCCCACCTGGCCAACCCCGTGCTGCCCAACGAAGTGCT|CCA**GGG**TG AGccccgaccccccgctgccccccagtcc
D312N-WT*	GCCGCCCGGGAGACGGACGCCCACCTGGCCAACCCCGTGCTGCCCGATGAAGTGCT|CCA**GGG**TG AGccccgaccccccgctgccccccagtcc
D609G-WT	GCCATCCTGCTGTCAGTGGCCCGGGGCAAAGTGTCCGAGGGAATCGA|CTT**TGG**TGAGTGGACTGGGC TCTTCCTCCCCAGGTGTCTTCCCAGA
D609G-MUT (for exon guide RNA)	GCCATCCTGCTGTCAGTGGCCCGGGGCAAAGTGTCCGAGGGAATCGG|CTT**TGG**TGAGTGGACTGGGC TCTTCCTCCCCAGGTGTCTTCCCAGA

<table>
<tr><td>D609G-WT* (for exon guide RNA)</td><td>GCCATCCTGCTGTCAGTGGCCCGGGGCAAAGTGTCCGAGGGAATCGA|TTT**TGG**TGAGTGGACTGGGC
TCTTCCTCCCCAGGTGTCTTCCCAGA</td></tr>
<tr><td>E606Q-WT</td><td>GGCCGCGGGGCCATCCTGCTGTCAGTGGCCCGGGGCAAAGTGTCCGAGGGAATCGA|CTT**TGG**TGAGT
GGACTGGGCTCTTCCTCCCCAGGTGT</td></tr>
<tr><td>E606Q-MUT</td><td>GGCCGCGGGGCCATCCTGCTGTCAGTGGCCCGGGGCAAAGTGTCCCAGGGAATTGA|CTT**TGG**TGAGT
GGACTGGGCTCTTCCTCCCCAGGTGT</td></tr>
<tr><td>E606Q-WT*</td><td>GGCCGCGGGGCCATCCTGCTGTCAGTGGCCCGGGGCAAAGTGTCCGAAGGAATTGA|CTT**TGG**TGAGT
GGACTGGGCTCTTCCTCCCCAGGTGT</td></tr>
<tr><td>N238S-WT (for exon guide RNA)</td><td>AAGGAACTGGCCCGCAAGGCCGTCGTGGTCTTCGACGAGGCCCACAA|CAT**TGG**TGAGGGGGGCGCC
AGGGGCCAAAGGGATGCCAGCCCCTCT</td></tr>
<tr><td>N238S-MUT (for exon guide RNA)</td><td>AAGGAACTGGCCCGCAAGGCCGTCGTGGTCTTCGACGAGGCCCACAG|CAT**TGG**TGAGGGGGGCGCC
AGGGGCCAAAGGGATGCCAGCCCCTCT</td></tr>
<tr><td>N238S-WT* (for exon guide RNA)</td><td>AAGGAACTGGCCCGCAAGGCCGTCGTGGTCTTCGACGAGGCCCACAA|TAT**TGG**TGAGGGGGGCGCCA
GGGGCCAAAGGGATGCCAGCCCCTCT</td></tr>
<tr><td>Q758E-WT</td><td>CAGCTAGAATCAGAGGAGACGCTGAAGAGGATAGAGCAGATTGCTCAGCAGCTCT|GAG**TGG**GG
CGGGTGGGGCCATAAACGGTTCCTGGTGAC</td></tr>
<tr><td>Q758E-MUT</td><td>CAGCTAGAATCAGAGGAGACGCTGAAGAGGATAGAGCAGATTGCTCAACAGCTGT|GAG**TGG**GG
CGGGTGGGGCCATAAACGGTTCCTGGTGAC</td></tr>
<tr><td>Q758E-WT*</td><td>CAGCTAGAATCAGAGGAGACGCTGAAGAGGATAGAGCAGATTGCTGAGCAGCTGT|GAG**TGG**GG
CGGGTGGGGCCATAAACGGTTCCTGGTGAC</td></tr>
<tr><td>S246Y-WT</td><td>CTGAGCCGGCTCTCTCCCCCTTCTCCAGACAACGTCTGCATCGACT**CCA**TGA|GCGTCAACCTCACCCGC
CGGACCCTTGACCGGTGCCAGGGC</td></tr>
<tr><td>S246Y-MUT</td><td>CTGAGCCGGCTCTCTCCCCCTTCTCCAGACAACGTCTGCATCGACT**ACA**TGA|GCGTCAACCTCACCCGC
CGGACCCTTGACCGGTGCCAGGGC</td></tr>
<tr><td>S246Y-WT*</td><td>CTGAGCCGGCTCTCTCCCCCTTCTCCAGACAACGTCTGCATCGACT**CGA**TGA|GCGTCAACCTCACCCGC
CGGACCCTTGACCGGTGCCAGGGC</td></tr>
<tr><td>T484M-WT</td><td>AAGATCCTGGACTTCCACCCCGTCACCATGGCAACCTTCACCATGA|CGC**TGG**CACGGGTCTGCCTCTGC
CCTATGGTGAGTGGGAGAGGCTAG</td></tr>
</table>

<table>
<tr><td>T484M-MUT</td><td>AAGATCCTGGACTTCCACCCCGTCACCATGGCAACCTTCACCATGA|TG**CTGG**CACGGGTCTGCCTCTGC
CCTATGGTGAGTGGGAGAGGCTAG</td></tr>
<tr><td>T484M-WT*</td><td>AAGATCCTGGACTTCCACCCCGTCACCATGGCAACCTTCACCATGA|CC**CTGG**CACGGGTCTGCCTCTGC
CCTATGGTGAGTGGGAGAGGCTAG</td></tr>
<tr><td>Y209C-WT</td><td>TCCCCGGCCCCCCAGATCCTGCATGCCAATGTGGTGGTTTATAGCTACCACTACC|TCC**TGG**ACCCC
AAGATTGCAGACCTGGTGTCCAAGGAA</td></tr>
<tr><td>Y209C-MUT</td><td>TCCCCGGCCCCCCAGATCCTGCATGCCAATGTGGTGGTTTATAGCTGCCACTATC|TCC**TGG**ACCCC
AAGATTGCAGACCTGGTGTCCAAGGAA</td></tr>
<tr><td>Y209C-WT*</td><td>TCCCCGGCCCCCCAGATCCTGCATGCCAATGTGGTGGTTTATAGCTATCACTATC|TCC**TGG**ACCCC
AAGATTGCAGACCTGGTGTCCAAGGAA</td></tr>
<tr><td>Y24C-WT</td><td>CTGGTCTACTTCCCGTACGACTACATCTACCCCGAGCAGTTCTCCTAC|ATG**CGG**GAGCTCAAACGCACG
CTGGACGCCAAGGTGGGTGGCCGG</td></tr>
<tr><td>Y24C-MUT</td><td>CTGGTCTACTTCCCGTACGACTACATCTACCCCGAGCAGTTCTCCTGC|ATG**CGG**GAGCTCAAACGCACG
CTGGACGCCAAGGTGGGTGGCCGG</td></tr>
<tr><td>Y24C-WT*</td><td>CTGGTCTACTTCCCGTACGACTACATCTACCCCGAGCAGTTCTCCTAT|ATG**CGG**GAGCTCAAACGCACG
CTGGACGCCAAGGTGGGTGGCCGG</td></tr>
<tr><td>Y639*-WT</td><td>ACCTCCCCAGCTTCTCATCCTCCGTATCTGCAGGCGCGGCTGGAATAC|CTG**CGG**GACCAGTTCCAGATT
CGTGAGAATGACTTTCTTACCTTC</td></tr>
<tr><td>Y639*-MUT</td><td>ACCTCCCCAGCTTCTCATCCTCCGTATCTGCAGGCGCGGCTGGAATAA|CTG**CGG**GACCAGTTCCAGATT
CGTGAGAATGACTTTCTTACCTTC</td></tr>
<tr><td>Y639*-WT*</td><td>ACCTCCCCAGCTTCTCATCCTCCGTATCTGCAGGCGCGGCTGGAATAT|CTG**CGG**GACCAGTTCCAGATT
CGTGAGAATGACTTTCTTACCTTC</td></tr>
<tr><td>F193V-WT</td><td>GATGACCTGAAGGCCCTGGGGCGGCGCCAGGGCTGGTG**CCC**ATA|CTTCCTTGCTCGATACTCAGTGAG
GAGGCTGGTGGGATGGCAGAGGGG</td></tr>
<tr><td>F193V-WT*</td><td>GATGACCTGAAGGCCCTGGGGCGGCGCCAGGGCTGGTGT**CC**ATA|CTTTCTTGCTCGATACTCAGTGAG
GAGGCTGGTGGGATGGCAGAGGGG</td></tr>
<tr><td>F193V-MUT</td><td>GATGACCTGAAGGCCCTGGGGCGGCGCCAGGGCTGGTGT**CC**ATA|CGTCCTTGCTCGATACTCAGTGA
GGAGGCTGGTGGGATGGCAGAGGGG</td></tr>
</table>

N250T-WT	TCTCCCCCTTCTCCAGACAACGTCTGCATCGACTCCATGAGCGTCAACCTCACC|CGC**CGG**ACCCTTGACC GGTGCCAGGGCAACCTGGAGACC
N250T-WT*	TCTCCCCCTTCTCCAGACAACGTCTGCATCGACTCCATGAGCGTCAATCTCACG|CGC**CGG**ACCCTTGAC CGGTGCCAGGGCAACCTGGAGACC
N250T-MUT	TCTCCCCCTTCTCCAGACAACGTCTGCATCGACTCCATGAGCGTCACCCTCACG|CGC**CGG**ACCCTTGAC CGGTGCCAGGGCAACCTGGAGACC
N238S-WT (for intron guide RNA)	AAGGAACTGGCCCGCAAGGCCGTCGTGGTCTTCGACGAGGCCCACAACATTGGTGAGGGGGGCG|CCA **GGG**GCCAAAGGGATGCCAGCCCCTCTGAGTGAGGCCCCTGCAGGCC
N238S-WT* (for intron guide RNA)	AAGGAACTGGCCCGCAAGGCCGTCGTGGTCTTCGACGAGGCCCACAA**T**ATTGGTGAGGGGGGCG|CG A**GGG**GCCAAAGGGATGCCAGCCCCTCTGAGTGAGGCCCCTGCAGGCC
N238S-MUT (for intron guide RNA)	AAGGAACTGGCCCGCAAGGCCGTCGTGGTCTTCGACGAGGCCCACAGCATTGGTGAGGGGGGCG|CG A**GGG**GCCAAAGGGATGCCAGCCCCTCTGAGTGAGGCCCCTGCAGGCC
D609G-WT (for intron guide RNA)	GCCATCCTGCTGTCAGTGGCCCGGGGCAAAGTGTCCGAGGGAATCGACTTTGGTGAGTGGACTGGGCT CTT**CCT**C**|**CCCAGGTGTCTTCCCAGA
D609G-WT* (for intron guide RNA)	GCCATCCTGCTGTCAGTGGCCCGGGGCAAAGTGTCCGAGGGAATCGATTTTGGTGAGTGGACTGGGCT CTT**CGT**C**|**CCCAGGTGTCTTCCCAGA
D609G-MUT (for intron guide RNA)	GCCATCCTGCTGTCAGTGGCCCGGGGCAAAGTGTCCGAGGGAATCGGCTTTGGTGAGTGGACTGGGCT CTT**CGT**C**|**CCCAGGTGTCTTCCCAGA

Supplemental Table 6: PCR primer sequences for first round of PCR. The adaptors required for the second round of PCR are indicated in blue.

**Name**	**Primer sequence (5' to 3')**
D179H-FWD	ACACTCTTTCCCTACACGACGCTCTTCCGATCTTGGCACTCTTGTCCCAGGA
D179H-REV	TGACTGGAGTTCAGACGTGTGCTCTTCCGATCTGCAACAGACAGACATGGGCA
D312N-FWD	ACACTCTTTCCCTACACGACGCTCTTCCGATCTCGCAGGATCAAAGAGACAGACG
D312N-REV	TGACTGGAGTTCAGACGTGTGCTCTTCCGATCTAGCCCAGGAAATGCTCGG
D609G/E606Q-FWD	ACACTCTTTCCCTACACGACGCTCTTCCGATCTAACTTGCGTCTCGGTCTCC
D609G/E606Q-REV	TGACTGGAGTTCAGACGTGTGCTCTTCCGATCTCTAGGGACAGAGGGGAGGG
D609G-FWD2	ACACTCTTTCCCTACACGACGCTCTTCCGATCTCCTCAGGCCTGCGAGAAT
D609G-REV2	TGACTGGAGTTCAGACGTGTGCTCTTCCGATCTGGGGAGAGGGTGTGTTCC
N238S-FWD	ACACTCTTTCCCTACACGACGCTCTTCCGATCTCCAAGATTGCAGACCTGGTG
N238S-RED	TGACTGGAGTTCAGACGTGTGCTCTTCCGATCTTGAGGTTGACGCTCATGGAG
N238S-FWD2	ACACTCTTTCCCTACACGACGCTCTTCCGATCTCCAAGATTGCAGACCTGGTG
N238S-REV2	TGACTGGAGTTCAGACGTGTGCTCTTCCGATCTGACGTTGTCTGGAGAAGGGG
Q758E-FWD	ACACTCTTTCCCTACACGACGCTCTTCCGATCTCCCTCTCCCTTTCCTCTGTT
Q758E-REV	TGACTGGAGTTCAGACGTGTGCTCTTCCGATCTAAGACCTTCTAGCACCACCG
S246Y/F-FWD	ACACTCTTTCCCTACACGACGCTCTTCCGATCTCTTCGACGAGGCCCACAAC
S246Y/F-REV	TGACTGGAGTTCAGACGTGTGCTCTTCCGATCTCCTGAGCACCGTCTTCTGC
T484M-FWD	ACACTCTTTCCCTACACGACGCTCTTCCGATCTGCTGGACATCTACCCCAAGA
T484M-REV	TGACTGGAGTTCAGACGTGTGCTCTTCCGATCTgcctcttcgctgtaAAGCTC
Y209C-FWD	ACACTCTTTCCCTACACGACGCTCTTCCGATCTggggtaggggtaggggtt
Y209C-REV	TGACTGGAGTTCAGACGTGTGCTCTTCCGATCTTTGTGGGCCTCGTCGAAG
Y24C-FWD	ACACTCTTTCCCTACACGACGCTCTTCCGATCTGCTGAGGGGACGGGAACT
Y24C-REV	TGACTGGAGTTCAGACGTGTGCTCTTCCGATCTCCCAGGGACCTCGGACTT
Y639*FWD	ACACTCTTTCCCTACACGACGCTCTTCCGATCTACACACAGAGCCGCATTCT

Y639*-REV	TGACTGGAGTTCAGACGTGTGCTCTTCCGATCTCCATGAGGCCGTAGTCCG
F193V-FWD	ACACTCTTTCCCTACACGACGCTCTTCCGATCTGTCCCAGGAATTTGATGCCC
F193V-REV	TGACTGGAGTTCAGACGTGTGCTCTTCCGATCTAACAGACAGACATGGGCAGA
N250T-FWD	ACACTCTTTCCCTACACGACGCTCTTCCGATCTGACAACGTCTGCATCGACTC
N250T-REV	TGACTGGAGTTCAGACGTGTGCTCTTCCGATCTGGGACAAGTCAGACAGGGG

References

1. Vokes NI, Liu D, Ricciuti B, Jimenez-Aguilar E, Rizvi H, Dietlein F, et al. Harmonization of Tumor Mutational Burden Quantification and Association With Response to Immune Checkpoint Blockade in Non–Small-Cell Lung Cancer. JCO Precis Oncol. 2019 Dec;(3):1–12.

2. Wang K, Li M, Hakonarson H. ANNOVAR: functional annotation of genetic variants from high-throughput sequencing data. Nucleic Acids Res [Internet]. 2010 Sep 1;38(16):e164–e164. Available from: https://academic.oup.com/nar/article-lookup/doi/10.1093/nar/gkq603

3. Cagiada M, Bottaro S, Lindemose S, Schenstrøm SM, Stein A, Hartmann-Petersen R, et al. Discovering functionally important sites in proteins. Nat Commun [Internet]. 2023 Jul 13;14(1):4175. Available from: https://www.nature.com/articles/s41467-023-39909-0

4. Cagiada M, Jonsson N, Lindorff-Larsen K. Decoding molecular mechanisms for loss of function variants in the human proteome. bioRxiv [Internet]. 2024 Jan 1;2024.05.21.595203. Available from: http://biorxiv.org/content/early/2024/05/22/2024.05.21.595203.abstract

5. Rives A, Meier J, Sercu T, Goyal S, Lin Z, Liu J, et al. Biological structure and function emerge from scaling unsupervised learning to 250 million protein sequences. Proc Natl Acad Sci [Internet]. 2021 Apr 13;118(15). Available from: https://pnas.org/doi/full/10.1073/pnas.2016239118

6. Hsu C, Verkuil R, Liu J, Lin Z, Hie B, Sercu T, et al. Learning inverse folding from millions of predicted structures. bioRxiv [Internet]. 2022 Jan 1;2022.04.10.487779. Available from: http://biorxiv.org/content/early/2022/09/06/2022.04.10.487779.abstract

7. Niu Y, Ferreira Azevedo CA, Li X, Kamali E, Haagen Nielsen O, Storgaard Sørensen C, et al. Multiparametric and accurate functional analysis of genetic sequence variants using CRISPR-Select. Nat Genet [Internet]. 2022 Dec 5;54(12):1983–93. Available from: https://www.nature.com/articles/s41588-022-01224-7
