# Supplementary material for: Quantitative functional profiling of ERCC2 mutations deciphers cisplatin sensitivity in bladder cancer
Source: J Clin Invest. 2025 Aug 15;135(16):e186688. doi: 10.1172/JCI186688 (PMC12352908; doi:10.1172/JCI186688)
Supplement: Unedited blot and gel images [file jci-135-186688-s235.pdf]

Supplemental Figure 3E

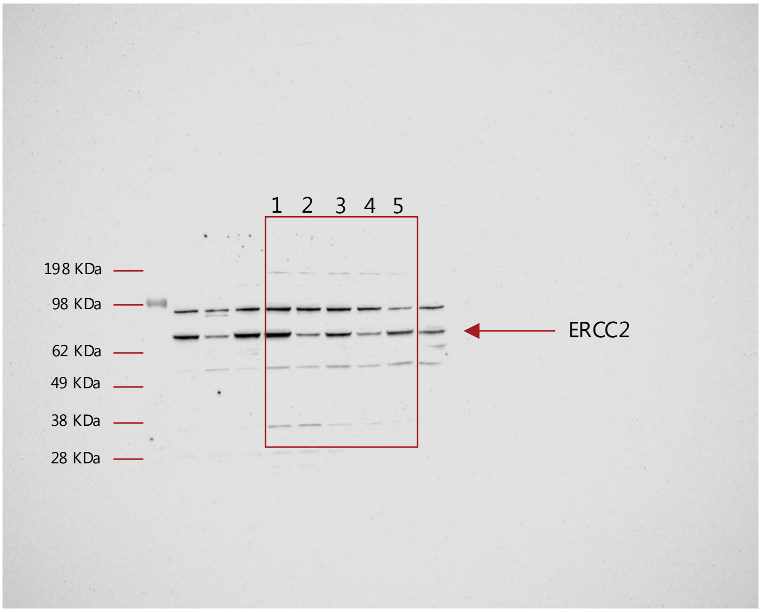

Lane 1: Non-targeting guide RNA
Lane 2: N238S Exon guide RNA
Lane 3: N238S Intron guide RNA
Lane 4: D609G Exon guide RNA
Lane 5: D609G Intron guide RNA

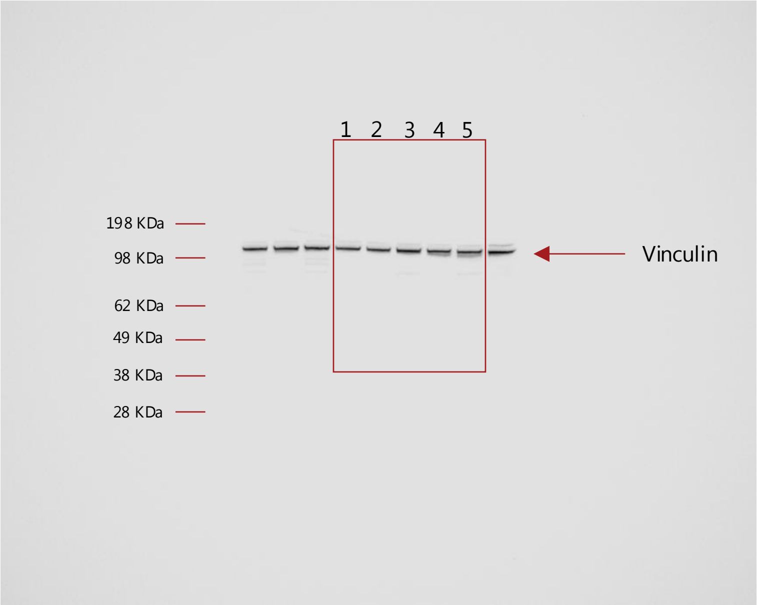

Lane 1: Non-targeting guide RNA
Lane 2: N238S Exon guide RNA
Lane 3: N238S Intron guide RNA
Lane 4: D609G Exon guide RNA
Lane 5: D609G Intron guide RNA
